# Classification of Volatile Organic Compounds Using a Novel High-Frequency Quartz Crystal Microbalance Sensor Array Based on Ethyl Cellulose Microstructures

**DOI:** 10.3390/s26185934

**Published:** 2026-09-19

**Authors:** Ian Chuey-Mendoza, Severino Muñoz-Aguirre, Isis I. Ramírez-Valdés, Claudia Mendoza-Barrera, Juan Castillo-Mixcóatl, Georgina Beltrán-Pérez, Marcos Rodríguez-Torres, Víctor Altuzar

**Affiliations:** Facultad de Ciencias Físico-Matemáticas, Benemérita Universidad Autónoma de Puebla, Avenida San Claudio y 18 Sur, Colonia San Manuel, Edificio FM1-101B, Ciudad Universitaria, Puebla C.P. 72570, Mexico; ian.chueym@gmail.com (I.C.-M.); smunoz@fcfm.buap.mx (S.M.-A.); isis.valdes@outlook.com (I.I.R.-V.); cmendoza@fcfm.buap.mx (C.M.-B.); jcastill@fcfm.buap.mx (J.C.-M.); gbeltran@fcfm.buap.mx (G.B.-P.); rodrigueztorresmarcos@gmail.com (M.R.-T.)

**Keywords:** ethyl cellulose, VOCs, high-frequency QCMs, sensor array, electrospray, multivariate analysis

## Abstract

Detecting volatile organic compounds (VOCs) is essential because they affect air quality and can serve as biomarkers for non-invasive early disease detection. VOCs can be identified using electronic noses combining cross-reactive sensors, pattern recognition, and classification algorithms. Here, a single-polymer 12-sensor array based on 30 MHz quartz crystal microbalances was developed using three ethyl cellulose (EC) microstructures: casting films (CFs), anti-solvent microparticles (ASMs), and electrospray microparticles (ESMs). The array detected ethanol, ethyl acetate, and heptane at ppm concentrations under controlled conditions of 20 °C and 20% relative humidity. Scanning electron micrographs exhibited average mesh-hole diameters of 1.13 ± 0.44 μm for CFs, and average particle diameters of 60 ± 15 nm and 1.1 ± 0.3 μm for ASMs and ESMs, respectively. FTIR spectra indicated O–H and H–O–H bending bands for ASMs and EC, whereas these bands were notably attenuated for ESMs, suggesting their affinities for polar and non-polar compounds, respectively. Mahalanobis distance and a support vector machine classifier accurately discriminated VOCs in principal component analysis space. Principal component regression and partial least squares regression predicted VOC concentrations with $R^{2}$ > 0.99, achieving estimated limits of detection of 143.6, 57, and 42.1 ppm by PCR and 70.5, 32.8, and 26.5 ppm by PLSR for ethanol, ethyl acetate, and heptane, respectively.

## 1. Introduction

Volatile organic compounds (VOCs) are involved in atmospheric photochemical reactions [1]. Their sources are anthropogenic (such as smoking, cutting grass, painting buildings, and cooking, among others) or biogenic [1,2,3,4]. Examples of VOCs include xylene, toluene, benzene, formaldehyde, ethanol, and acetic acid, which significantly impact ecosystems and human health [2,3,4,5]. The oxidation of VOCs in the presence of nitrogen oxides (NO_x_) and sunlight contributes to the formation of tropospheric ozone ($O_{3}$), especially in urban and industrial areas, and adds to global warming. Both VOCs and $O_{3}$ require continuous monitoring because they are hazardous to humans, causing eye and nose irritation, respiratory diseases, impaired lung function, asthma, oxidative cellular damage, immune-inflammatory responses, multiple types of cancer, and reproductive risks [3,4,6,7,8,9,10]. Human breath contains more than 100 VOC types [1,2]. Changes in these VOCs can serve as potential biomarkers for the non-invasive, early diagnosis of various diseases [11,12,13,14,15,16]. Patients with lung cancer exhale hydrocarbons such as pentane, heptane, toluene, and isoprene, as well as primary and secondary alcohols, including 1-propanol [12,15]. Individuals with type 1 diabetes exhale acetone at levels over 1800 ppb, compared with 300–900 ppb in healthy individuals [12,16].

Gas chromatography–mass spectrometry (GC–MS) is widely used to measure VOCs in the atmosphere and human breath, detecting thousands of compounds. Although precise, it requires bulky equipment and trained personnel, making the analysis time-consuming and unsuitable for real-time measurements. Alternatives include field-effect transistor (FET) sensors, surface acoustic wave (SAW) sensors, electrochemical (ES) sensors, quartz crystal microbalance (QCM) sensors, and others. QCMs are low-cost gravimetric devices based on quartz crystal wafers that measure VOCs by monitoring changes in the coated electrode’s mass. Changes in resonance frequency indicate the presence of VOCs in real time, surpassing the capabilities of MS [2,17,18]. To detect a specific VOC or a collection of VOCs, electrodes are chemically modified with metal oxides, carbon-based materials, ionic liquids, metal–organic frameworks (MOFs), or polymers. VOCs have a strong affinity for polymers via intermolecular interactions and adsorption sites, offering partial selectivity [2,19,20]. Their viscoelastic nature and relative ease of synthesis enable diverse structures through different deposition techniques [19]. Nanostructured polymers with higher surface-area-to-volume ratios provide increased sensitivity, faster response/recovery, and selectivity for specific compounds compared with their film counterparts [21,22]. Applications include sensing formaldehyde with styrene-methacrylic-acid nanoparticles under challenging environmental conditions [23], improved sensitivity to ammonia, methanol, acetonitrile, and acetic acid by nanoparticle size reduction [24], and rapid, reversible HCl detection with poly(N-isopropyl acrylamide) nanoparticles, outperforming slower film coatings [25].

Ethanol is widely used in large-scale industries [26] and remains underexplored as an oxygenated volatile organic compound (VOC) in the atmosphere [27]. Ethyl acetate is associated with secondary anemia and liver function impairment and serves as a biomarker for colorectal and gastric cancers in human breath [28]. Heptane is recognized as an environmental pollutant, as an industrial solvent [29], and as a potential indicator of lung cancer in human breath [13]. Ethanol, ethyl acetate, and heptane were selected as representative VOCs of the alcohol, ester, and alkane classes. These compounds exhibit high, medium, and low polarities, as well as distinct Hansen solubility parameters.

Because VOC mixtures, rather than single compounds, are common in the atmosphere and in human breath, cross-reactive sensing with electronic noses is crucial. This approach mimics the biological nose’s sense of smell, using sensor arrays with different coatings to detect compounds and pattern-recognition methods to generate response fingerprints. Used in disease detection and food monitoring, these versatile tools incorporate nanomaterials as coatings, significantly enhancing their performance [2].

Electro-hydrodynamic atomization, or electrospray, is a versatile method for fabricating particles with diverse compositions and controllable structures, sizes, and shapes. This enables precise deposition of micro- and nanostructures. Its popularity in drug delivery stems largely from the simplicity and flexibility of its experimental setup. The system typically includes a syringe pump, a metal needle, a high-voltage supply, and a grounded substrate that collects the deposited particles. A solution is pumped through the needle, positioned at a defined distance from the collector, and a high voltage is applied to both the needle and the grounded collector. This creates a Taylor cone as electrostatic and capillary forces interact. When electrostatic pressure exceeds capillary pressure, it overcomes the surface tension of the polymer solution, forming droplets that travel to the collector. Electrospray is widely used in microelectronics, sensing, chemical analysis, drug delivery, pharmaceuticals, and food processing. It is useful for surface doping, coating, and modification to enhance sensitivity and selectivity [30,31,32,33,34].

The novelty of this study is the development of a VOC sensor array comprising three distinct microstructures derived from a single polymer, ethyl cellulose. Unlike many arrays that use multiple polymers, the proposed 12-sensor array enables VOC classification and concentration prediction using microstructural diversity within a single polymer [35,36,37,38,39]. In other words, a simple and effective single-polymer 12-sensor array was developed for the first time in this study. In particular, the array consists of twelve 30 MHz quartz crystal microbalances (QCMs) coated with those three ethyl cellulose microstructures, four QCMs per type. These microstructures are (1) ethyl cellulose casting films (CFs) [40,41,42,43,44] applied by drop-casting; (2) ethyl cellulose anti-solvent microparticles (ASMs) [45], also applied by drop-casting; and (3) electrospray ethyl cellulose microparticles (ESMs). This sensor array was employed to detect ethanol, ethyl acetate, and heptane at various parts-per-million concentrations. Principal component regression and partial least squares regression predicted VOC concentrations with $R^{2}$ > 0.99, achieving estimated limits of detection of 143.6, 57, and 42.1 ppm by PCR and 70.5, 32.8, and 26.5 ppm by PLSR for ethanol, ethyl acetate, and heptane, respectively.

## 2. Theory

### 2.1. Sauerbrey Equation

The QCM works by the reverse piezoelectric effect. When a voltage is applied, the crystal deforms. Specifically, applying an alternating current (AC) voltage across the electrodes of an AT-cut quartz crystal induces thickness-shear deformation. This motion generates acoustic waves in the crystal, causing the entire crystal to oscillate. The maximum oscillation amplitude occurs when the AC voltage frequency f matches the fundamental resonance frequency $f_{0}$ of the crystal. The Sauerbrey Equation, Equation (1), relates the frequency shift Δf at $f_{0}$ to the change in mass Δm at the surface of the electrodes. It assumes that mass sensitivity is constant across the electrodes’ surface [46,47,48].

(1) $$\Delta f=-\frac{2f_{0}^{2}}{\sqrt{\rho_{q}{\overset{-}{c}}_{66}}}\frac{\Delta m}{A_{s}}=-C_{\mathrm{QCM}}\Delta m,\;\;\;\mathrm{where}\;\;C_{\mathrm{QCM}}=\frac{2f_{0}^{2}}{\sqrt{\rho_{q}{\overset{-}{c}}_{66}}A_{s}},$$

where $\rho_{q}$ is the density of quartz, $A_{s}$ is the effective electrode surface area, and ${\overset{-}{c}}_{66}$, Equation (2), is the piezoelectrically stiffened shear modulus,

(2) $${\overset{-}{c}}_{66}=c_{66}+\frac{e_{26}^{2}}{\varepsilon_{22}},$$

where $c_{66}$ is the elastic constant, $e_{26}$ is the piezoelectric constant, and $\varepsilon_{22}$ is the dielectric constant of the AT-cut quartz crystal, and $C_{\mathrm{QCM}}$ (Hz/kg) is the Sauerbrey mass sensitivity of the QCM [49,50]. Then, the effective thickness $t_{o}$ of a sensitive coating on the QCM can be expressed as Equation (3),

(3) $$t_{o}=-\frac{\sqrt{\rho_{q}{\overset{-}{c}}_{66}}}{2f_{0}^{2}\rho_{c}}\Delta f,$$

where $\rho_{c}$ is the density of the coating material. We use effective thickness because Equation (3) is equivalent to the measure of mass loading over the electrodes, and it is not a measure of the real thickness of the coating.

Although Equation (1) is a valid approximation and one we used for sensor comparisons, it does not accurately reflect the distribution of mass sensitivity across the electrode surface. Mass sensitivity depends on electrode shape and size and varies with location on the crystal and electrode surface. Huang et al. [50] present the sensitivity function distributions for 5 MHz and 10 MHz QCMs. In their analysis, the electrodes are circular and have the same radius. The red region indicates the peak in mass sensitivity, which decreases toward the edges. This observation has practical implications for how volatile organic compounds (VOCs) interact with the coating as they transition from the electrode to the non-electrode region. Accordingly, when the coating covers the entire electrode, edge interactions contribute less to changes in fundamental frequency than center interactions, which directly connects this observation to the sensitivity trend.

As will be shown in Section 4.2, sensor sensitivity increases linearly with effective thickness, or mass loading, for each ethyl cellulose microstructure type, regardless of its distribution on the electrodes. These results more directly support the applicability of the Sauerbrey equation under the experimental conditions used in this work.

### 2.2. VOC Concentration Equation

The concentration of VOCs within the measurement chamber of the static system was quantified in parts per million (ppm) according to Equation (4),

(4) $$C_{\mathrm{ppm}}=\frac{22.4T_{a}\rho_{s}V_{l}}{273.15M_{w}V_{c}},\;$$

where $C_{\mathrm{ppm}}$ is the VOC concentration (ppm), 22.4 L denotes the molar volume of an ideal gas at standard conditions (1 atm, 273.15 K), $T_{a}$ is the measurement temperature (K), $\rho_{s}$ is the VOC density (g/L), $V_{l}$ is the injected VOC volume (µL, liquid), $M_{w}$ is the VOC molar mass (g/mol), and $V_{c}$ is the chamber volume (1 L) [44].

Given that $V_{l}\ll V_{c}$, and the high vapor pressures of ethanol, ethyl acetate, and heptane at 20 °C, approximately 6.6, 10, and 6 kPa, respectively, these compounds are expected to completely vaporize under the experimental conditions. Notably, the vapor pressures are at least two orders of magnitude higher than 10 Pa, which is a vapor-pressure threshold reported by Koppmann [1] as one of the criteria used to classify a compound as a VOC. The vapor pressures at 20 °C were estimated using Antoine’s semi-empirical equation and the corresponding Antoine coefficients reported by Yaws [51].

### 2.3. Partition Coefficient

Van der Waals forces, polarity, and hydrogen bonding influence vapor sorption within the sorbent EC microstructures and, consequently, sensor selectivity. These interactions are quantified by the partition coefficient K, which represents the distribution of vapor between the gas and sorbent phases and encompasses all the relevant fundamental interactions. The partition coefficient is defined as the ratio of vapor concentration in the sorbent phase $C_{s}$ to that in the gas phase $C_{v}$, as shown in Equation (5),

(5) $$K=\frac{C_{s}}{C_{v}}.\;\;$$

The response of an acoustic wave sensor ${\Delta f}_{v}$, such as a QCM, to the mass of absorbed vapor is related to K, Equation (6), as

(6) $${\Delta f}_{v}=n\frac{{\Delta f}_{s}C_{v}K}{\rho_{s}},\;\;$$

where ${\Delta f}_{s}$ is the frequency shift of the sorbent (sensor element), $\rho_{s}$ is its density, and n is the amplification factor influenced by modulus changes due to swelling. According to Grate [52] and given that the response of our sensors is linear, we could assume that only mass loading occurs, n = 1, then  K is given by Equation (7),

(7) $$K=\frac{{\Delta f}_{v}\rho_{s}}{{\Delta f}_{s}C_{v}},\;$$

where $C_{v}$ could be reformulated as Equation (8),

(8) $$C_{v}=\frac{m_{v}}{V_{c}}=\frac{\rho_{v}V_{l}}{V_{c}}.\;$$

Then, K becomes Equation (9),

(9) $$K=\frac{{\Delta f}_{v}\rho_{s}V_{c}}{{\Delta f}_{s}\rho_{v}V_{l}},$$

where $V_{l}$ is the vapor volume to which the acoustic wave sensor was exposed [42,44,52]. Given that $V_{l}\ll V_{c}$, as stated in Section 2.2, and while the frequency change resulting from interaction between the VOC and the coating also increases linearly with VOC concentration, as discussed in Section 4.2, these observations directly support the assumption that no significant swelling occurs in the sorbent phase. This finding indicates that mass loadings are predominant within the range of effective thickness and VOC concentrations addressed in this paper; thus, our sensors can be considered as acoustically thin thickness-shear-mode devices [52,53]. Therefore, *n* can be approximated to 1.

## 3. Materials and Methods

### 3.1. Materials

The reagents used to prepare the ethyl cellulose casting films, anti-solvent microparticles, and electrospray microparticle sensor elements included ethyl cellulose with an ethoxyl content of 48% (EC, CAS 9004-57-3, 200646, Sigma-Aldrich, St. Louis, MO, USA), chloroform (TCM, CTR Scientific, CTR01876, Monterrey, Nuevo Leon, Mexico), acetone (ACT, CAS 67-64-1, Sigma-Aldrich, St. Louis, MO, USA), absolute ethanol (AEtOH, CAS 64-17-5, Karal, León, Guanajuato, Mexico), and deionized water (DW, CAS 7732-18-5, Karal, León, Guanajuato, Mexico). The VOCs included denatured ethanol (EtOH, CAS 64-17-5, 3014-03, J. T. Baker, Radnor, PA, USA), ethyl acetate (EA, CAS 141-78-6, 319902, Sigma-Aldrich, St. Louis, MO, USA), and heptane (hp, CAS 142-82-5, H2198, Sigma Aldrich, St. Louis, MO, USA). All reagents were analytical grade and used without further purification. The 30 MHz QCMs were purchased from MEC Crystals (Dongguan, China) and consisted of a 6 mm diameter AT-cut quartz crystal wafer with approximately 2 mm diameter electrodes. Table 1 summarizes the physicochemical properties of EC and the VOCs analyzed in this study.

### 3.2. Synthesis of Ethyl Cellulose Anti-Solvent Microparticles

Ethyl cellulose anti-solvent microparticles (ASMs) were synthesized using an anti-solvent particle-induction method [45]. First, 37.5 mg of EC was dissolved in 12.5 mL of ACT. Then, 50 mL of DW was added at 500 mL/h via a syringe pump (KDS 100, KDS Scientific, Holliston, MA, USA) using a 20 G needle positioned 27 cm above the surface, while stirring at 150 rpm for 1 h. After evaporating ACT for over 72 h, an ASM aqueous suspension at 0.75 mg/mL was obtained. The suspension was freeze-dried at −50 °C and 0.047 mbar for 12 h, and a 1 mg/mL ASM aqueous suspension was then prepared.

### 3.3. Construction of QCM Sensors

Twelve 30 MHz QCMs were used to fabricate QCM sensors with the three EC microstructures, with four QCMs per type (CF, ASM, or ESM). Each was opened by removing the metal casing with a rotary tool. Subsequently, a one-minute UV-ozone cleaning treatment (UV/Ozone ProCleaner™, BioForce Nanosciences, Inc., Ames, IA, USA) was applied to both sides to remove organic residues. Then, the QCM’s fundamental frequency $f_{0}$ was determined using a voltage divider circuit, a waveform generator (33600A, Keysight Technoligies, Santa Rosa, CA, USA), and an oscilloscope (DSOX4024A, Keysight Technologies, Santa Rosa, CA, USA). A 10 $V_{\mathrm{pp}}$ signal from 29.85 to 30.15 MHz was applied through a 1 kΩ resistor in series with the QCM and the QCM’s output voltage $V_{\mathrm{out}}$ was recorded. The minimum voltage occurs at the fundamental frequency $f_{0}$.

#### 3.3.1. Deposition of Ethyl Cellulose Films on QCM Sensors

**Figure 1:**
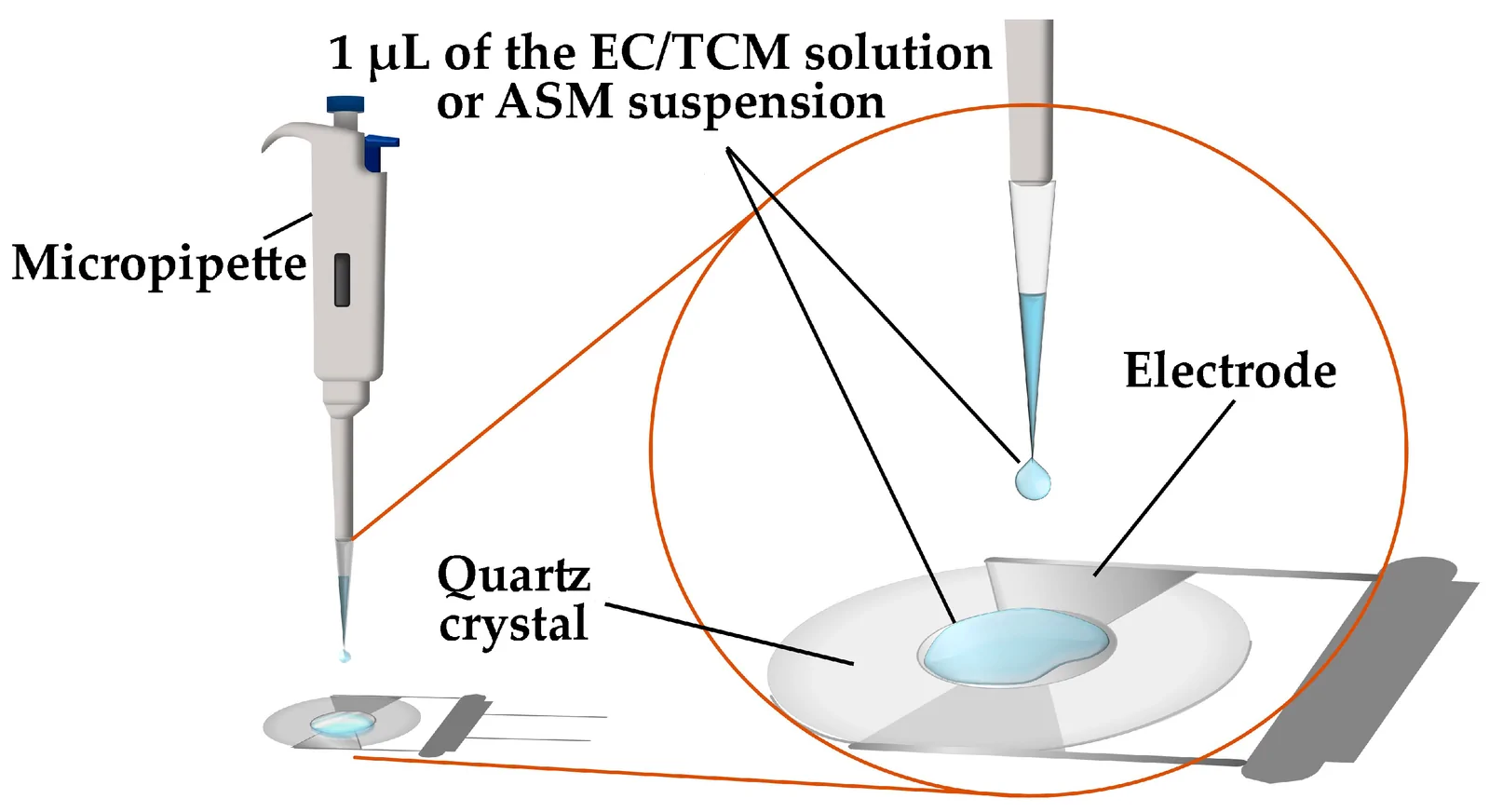
Experimental setup for drop-casting of the EC/TCM solution and ASM suspension.

Ethyl cellulose casting films (CFs) were prepared from an EC/TCM solution at 0.1% *w*/*v*. Then, 1 μL of the EC/TCM solution was deposited by drop-casting onto each electrode of four QCMs and left to evaporate for 10 min (Figure 1). A second sweep measured the new frequency $f_{f}$, and the frequency shift $\Delta f=f_{f}-f_{0}$ was used in Equation (3) to calculate the effective thickness $t_{o}$ of the CFs (Table 2). All sensor elements were degassed for 24 h before VOC testing.

#### 3.3.2. Deposition of Ethyl Cellulose Anti-Solvent Microparticles on QCM Sensors

On each electrode of the QCMs, 1 μL of the ASM suspension was deposited by drop-casting (Figure 1). The QCMs were then heated at 60 °C for 10 min to evaporate DW and promote self-assembly. After sealing with their metal casings, a second frequency sweep, $f_{f}$, was measured, and $\Delta f=f_{f}-f_{0}$ was used in Equation (3) to calculate the effective thickness $t_{o}$. The $t_{o}$ values and sensor labels are shown in Table 2. As before, all sensor elements were degassed for 24 h before VOC testing.

#### 3.3.3. Synthesis and Deposition of Ethyl Cellulose Electrospray Microparticles on QCM Sensors

**Figure 2:**
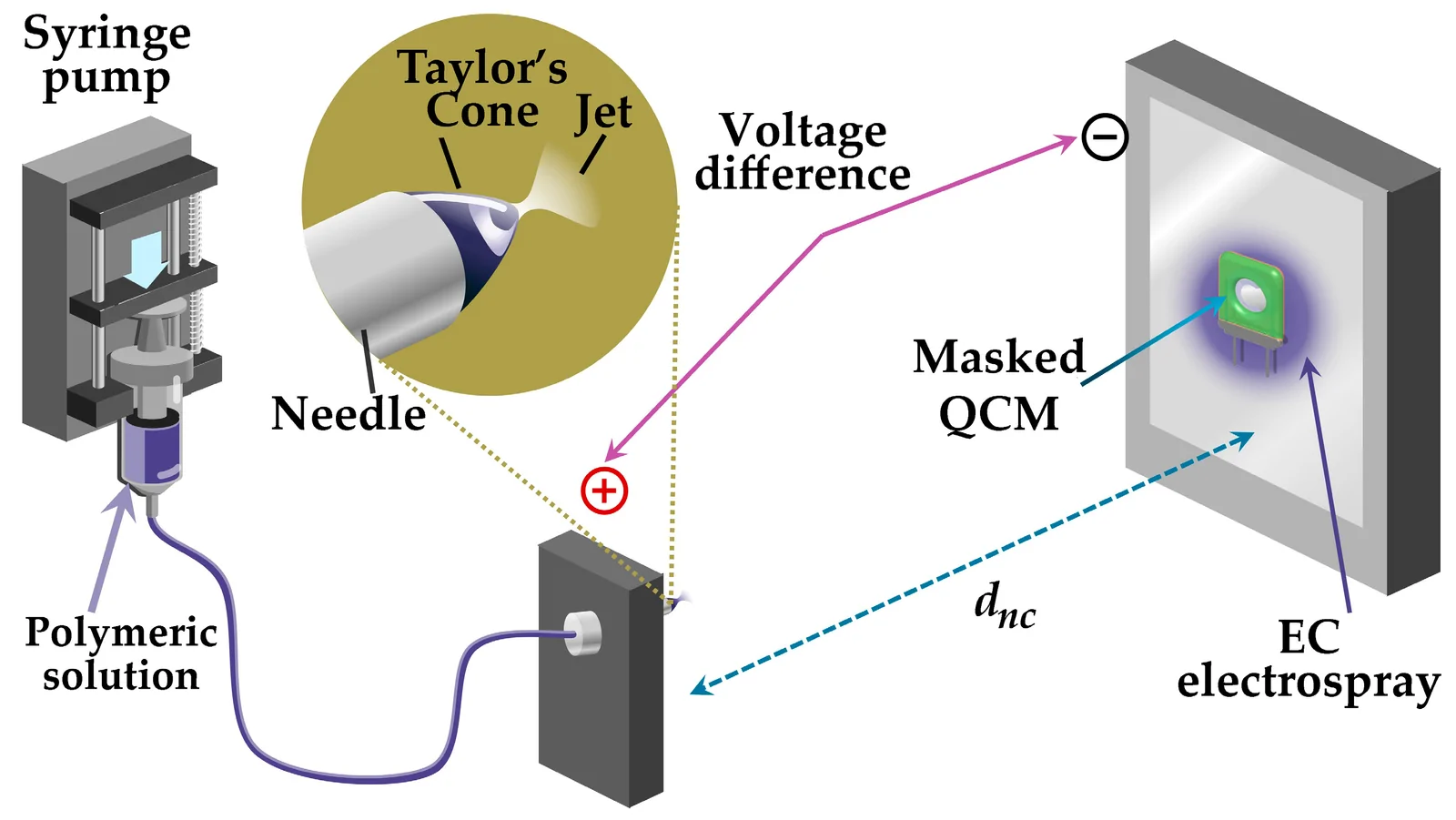
Experimental setup for the electrospray deposition of the EC/EtOH onto the QCMs.

A 0.3% *w*/*v* EC/EtOH solution was prepared by dissolving 15 mg of EC in 5 mL of absolute EtOH and stirring at 150 rpm for 1 min. The solution was loaded into a syringe pump of an electrospinning device (Fluidnatek LE-50, Bioinicia Fluidnatek, Paterna, Valencia, Spain). The solution was electrosprayed onto a fixed QCM at the collector center, covered with a resin mask that exposed only the 2 mm electrodes (Figure 2). At a needle-to-collector distance of 10 cm and an applied voltage of 13 kV, the solution was pumped at 0.5 mL/h, forming a Taylor cone and depositing EC electrospray on the electrodes. After 1 min, the voltage and pump were stopped, the mask was removed, and the QCM resealed. As before, the new fundamental frequency *f_f_* and Δf were determined, to calculate $t_{o}$ (Table 2). The procedure was repeated for another QCM at 10 cm and for two additional QCMs at $d_{\mathrm{nc}}$ = 5 cm. All sensor elements were degassed for 24 h before VOC testing.

### 3.4. Steady-State Sensor Response Measurement Process

**Figure 3:**
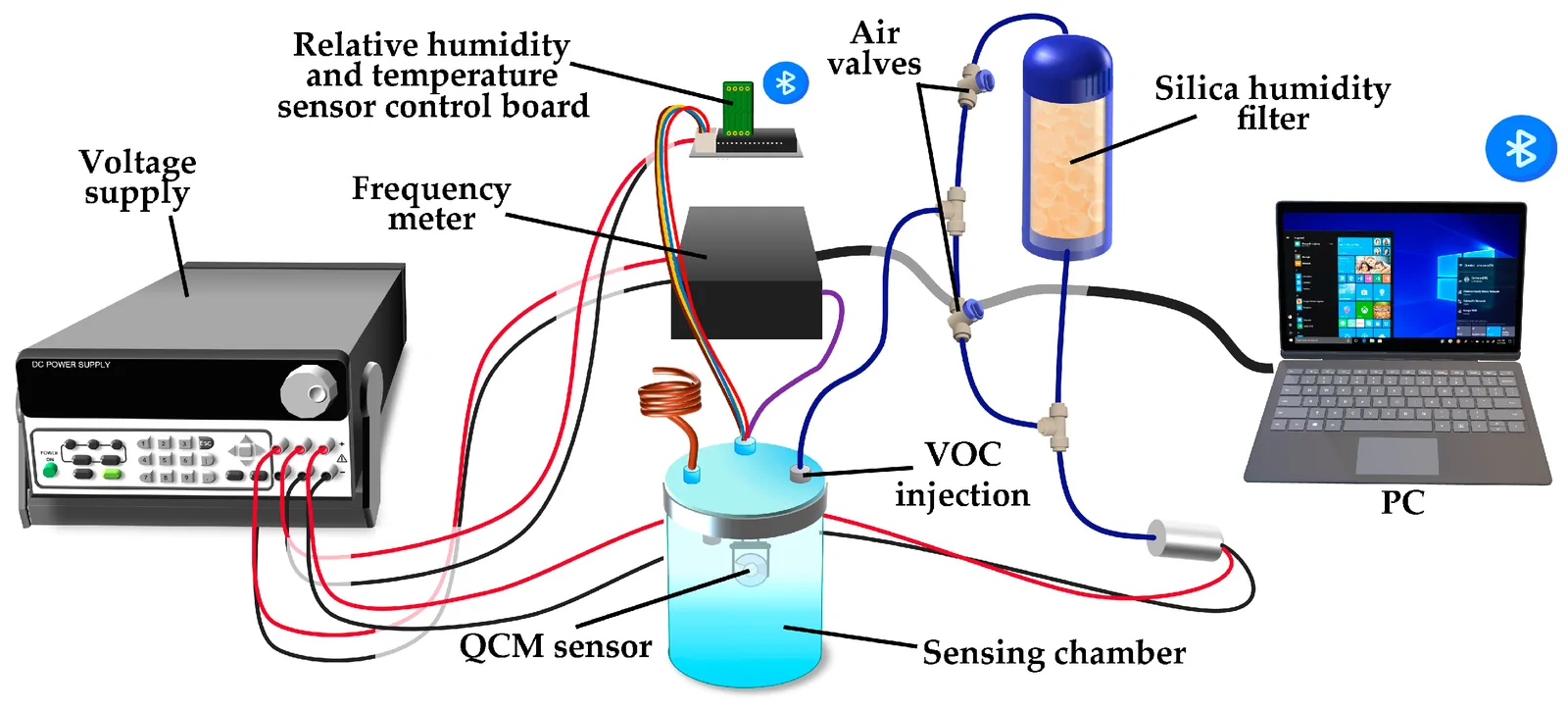
Experimental setup for the steady-state sensor response measurement using QCM sensors.

Figure 3 shows the setup for measuring the VOC steady-state response. The QCM sensor was connected to the oscillator circuit inside a sealed chamber, with baseline conditions of 20 °C and 20% RH maintained by an air pump and a silica filter. Then, 5 μL of denatured EtOH was injected, and the sensor frequency was monitored as it decreased and stabilized due to interaction with the EC microstructure. Humid air was introduced for 4 min to purge the chamber, raising RH to ~45% and decreasing the frequency; then the filter was reconnected, and RH was restored to 20%. This was repeated with 10 and 15 μL of EtOH four times. The same protocol was applied to EtOAc and hp. Table 3 lists the VOC concentrations in the chamber, calculated using Equation (4). The steady-state response measurement was repeated with all 12 sensors, each exposed to each VOC 4 times, for a total of 36 measurements per sensor.

### 3.5. Characterization of Ethyl Cellulose Microstructures

#### 3.5.1. Scanning Electron Microscopy Morphology Characterization Conditions

For SEM characterization, 1 μL of 0.1% *w*/*v* EC/TCM solution was deposited on one electrode of a 30 MHz QCM for the CF, and 1 μL of 0.1% *w*/*v* ASM suspension was deposited on a second QCM. For ESMs, a 0.3% *w*/*v* EC/EtOH solution was electrosprayed for 10 min at a $d_{\mathrm{nc}}$ = 10 cm onto a cleaned QCM without the resin mask, and the procedure was repeated with a 5% *w*/*v* EC/EtOH solution. Finally, the AT-cut quartz crystals were removed from their cases; the crystals were placed with the EC microstructures facing upward in plastic petri dishes and stored in a desiccator for 24 h before SEM imaging.

The CFs (at 5000× and 25,000×) and ESMs (at 2000× with both 0.3% m/v and 5% m/v solutions) were characterized using a field-emission Jeol JSM-7800F (JEOL Ltd., Akishima, Tokyo, Japan) and required no metallic coating; the ASMs (at 67×, 15,000×, and 48,700×) were characterized with a Jeol JSM-6610 (JEOL Ltd., Akishima, Tokyo, Japan) and were coated with gold. A total of 500 particles were measured in the electrospray of the 5% m/v solution, as well as for the inner and outer zones of the ASM droplet on the QCM electrode (a total of 1000 particles); 250 holes were measured in the CFs, and 100 particles were measured for the case of the 0.3% m/v ESM solution. Hole and particle size measurements were performed manually using the ImageJ software (version 1.54p). Because these microstructures were not perfect spheres or holes, the observed major and minor axes of each particle were averaged.

#### 3.5.2. Fourier Transform Infrared Spectroscopy of Ethyl Cellulose Microstructure Analysis

10 mg of ethyl cellulose, electrosprayed ethyl cellulose microparticles, and freeze-dried antisolvent ethyl cellulose microparticle powders were characterized via FTIR (Nicolet iS50 FT-IR spectrometer, Thermo Electron Scientific Instruments LLC, Madison, WI, USA) using the ATR technique. The functional groups in the normalized transmittance spectra of the three microstructures were analyzed to determine chemical structural modifications during the synthesis procedures.

#### 3.5.3. Multivariate Analysis of Steady-State Responses and Partition Coefficients

The steady-state responses and the natural logarithms of their partition coefficients were analyzed using principal component analysis (PCA) for feature extraction and dimensionality reduction, Mahalanobis distance, and support vector machine (SVM) for VOC classification, and principal component regression (PCR) and partial least squares regression (PLSR) for VOC concentration prediction and RMSE-based estimation of the limits of detection. The optimal number of principal components in PCR and latent variables in PLSR was determined by cross-validation.

## 4. Results and Discussion

### 4.1. Analysis of the Characterization of Ethyl Cellulose Microstructures

#### 4.1.1. Scanning Electron Microscopy Analysis

**Figure 4:**
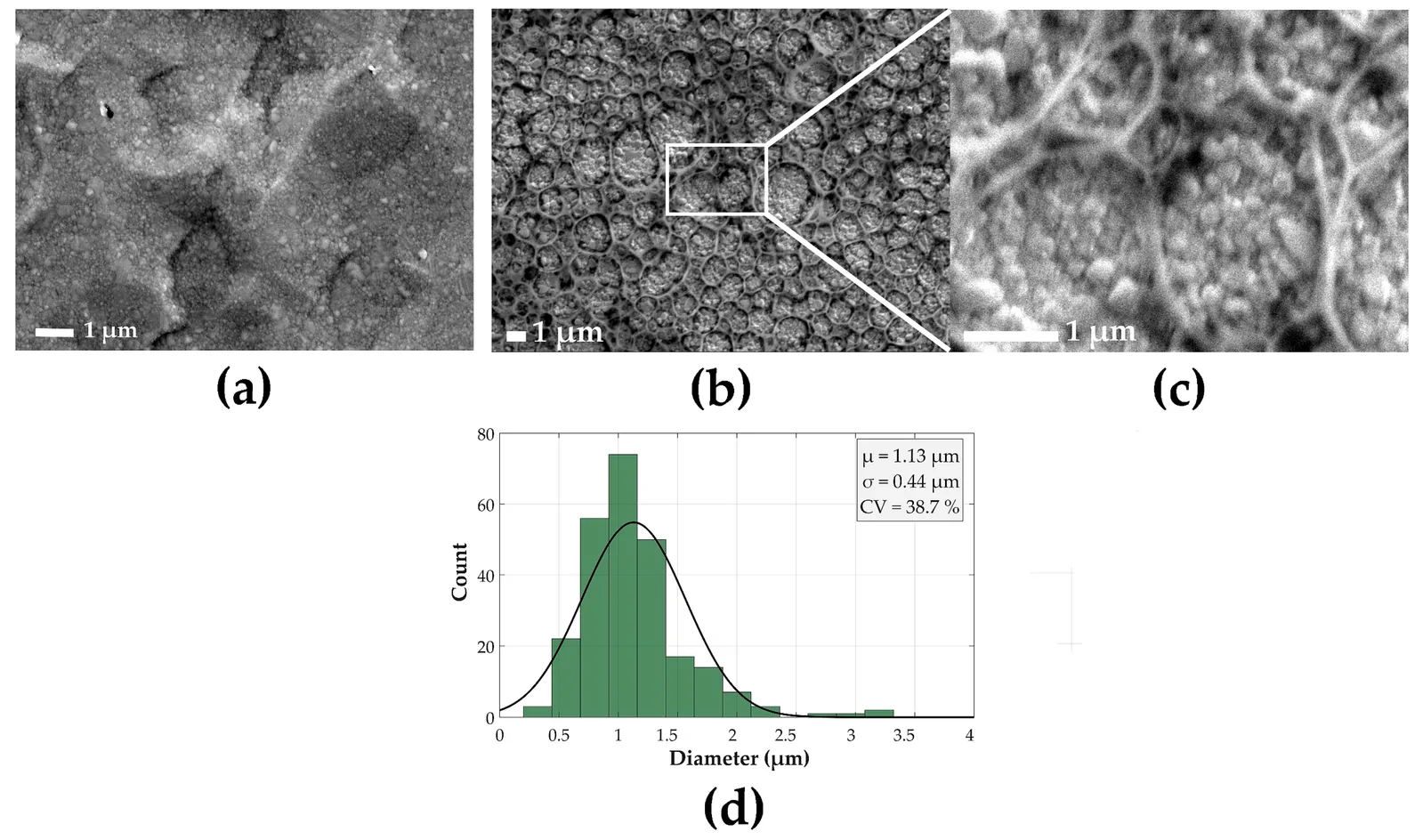
FE-SEM micrographs of (**a**) a bare silver electrode of a 30 MHz QCM at 10,000× magnification and a CF deposited on one electrode at (**b**) 5000× and (**c**) 25,000× magnification; (**d**) mesh-hole diameter distribution with mean diameter μ, standard deviation σ, and coefficient of variation CV.

The electrode surface of a 30 MHz QCM and the morphologies of the three types of EC microstructures deposited onto the 30 MHz QCMs SEM was used to characterize the morphologies of (1) CFs, (2) ASMs deposited by drop casting, and (3) ESMs on 30 MHz QCM electrodes. A micrograph of the bare silver-electrode surface is shown in Figure 4a, with a mean roughness of approximately 71.4 nm [43]. In addition to exhibiting a non-smooth topography characterized by valleys and peaks, the electrode surface contains grains resulting from the silver deposition process onto the quartz crystal.

In the case of the CF deposit, Figure 4 shows the formation of a mesh network with openings of different sizes (Figure 4b) on the QCM electrode substrate (Figure 4c). This type of morphology has been previously observed using atomic force microscopy [43]. The average diameter over 250 meshes was 1.13 ± 0.44 μm, with a coefficient of variation (CV) of 38.7% (Figure 4d).

**Figure 5:**
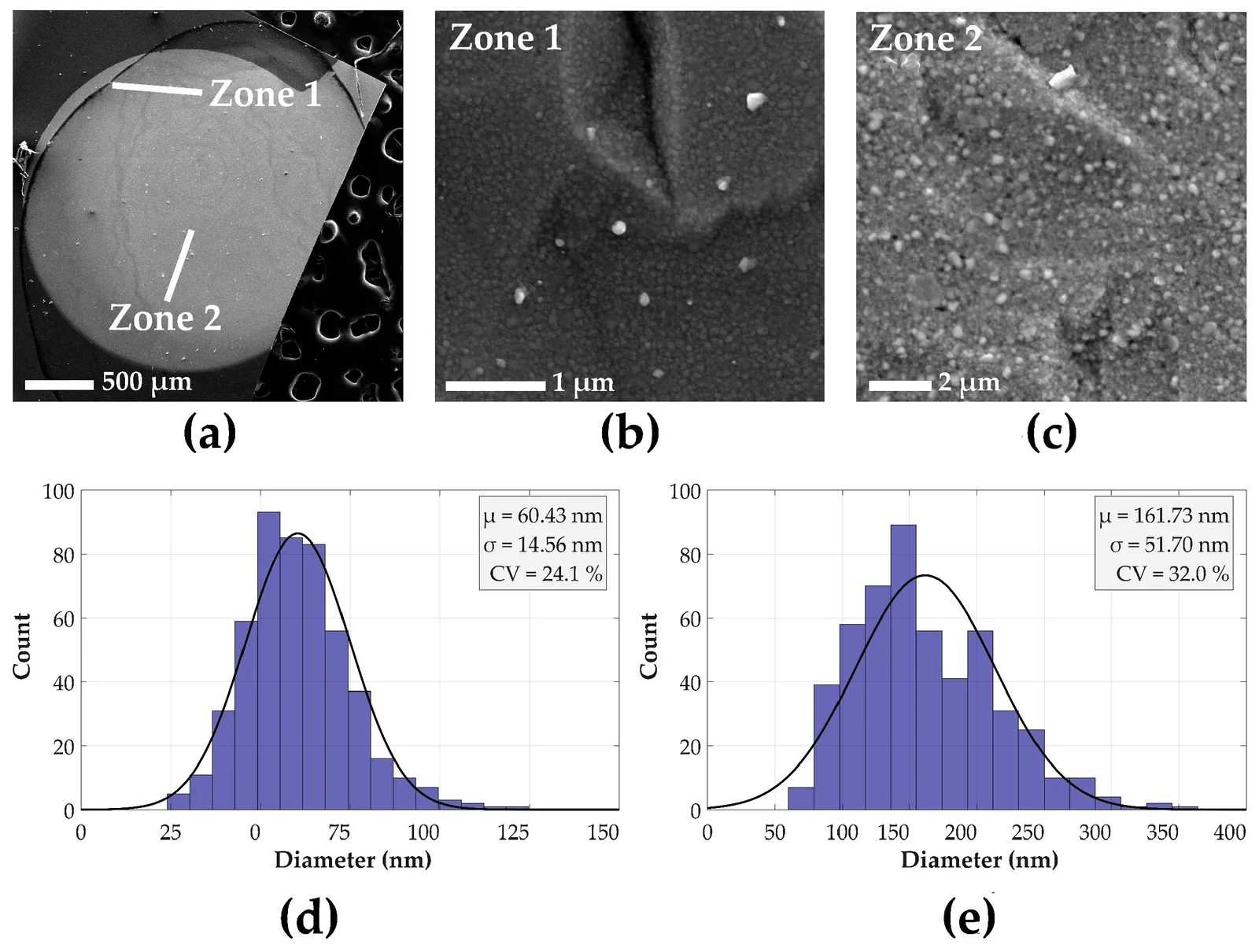
SEM micrographs of ASMs deposited on one electrode of a 30 MHz QCM: (**a**) overview at 67×, (**b**) Zone 1 at 48,700×, and (**c**) Zone 2 at 15,000× magnification. Particle-diameter distributions for (**d**) Zone 1 and (**e**) Zone 2 are shown with mean diameter μ, standard deviation σ, and coefficient of variation CV.

For the ASM sensing element fabricated by drop-casting, as shown in Figure 5, the characteristic coffee-ring effect is observed (Figure 5a). Zone 1 (Figure 5b) contains most of the smaller particles, with an average diameter of 60 ± 15 nm and a CV of 24% (Figure 5d). By comparison, at the center of the 30 MHz QCM, in Zone 2 (Figure 5c), larger particles with an average diameter of 162 ± 52 nm and a CV of 32% (Figure 5e) were deposited. This particle organization on the QCM electrode can be attributed to capillary flow induced by the evaporation of the water drop, which mobilizes smaller particles to the edge [62].

**Figure 6:**
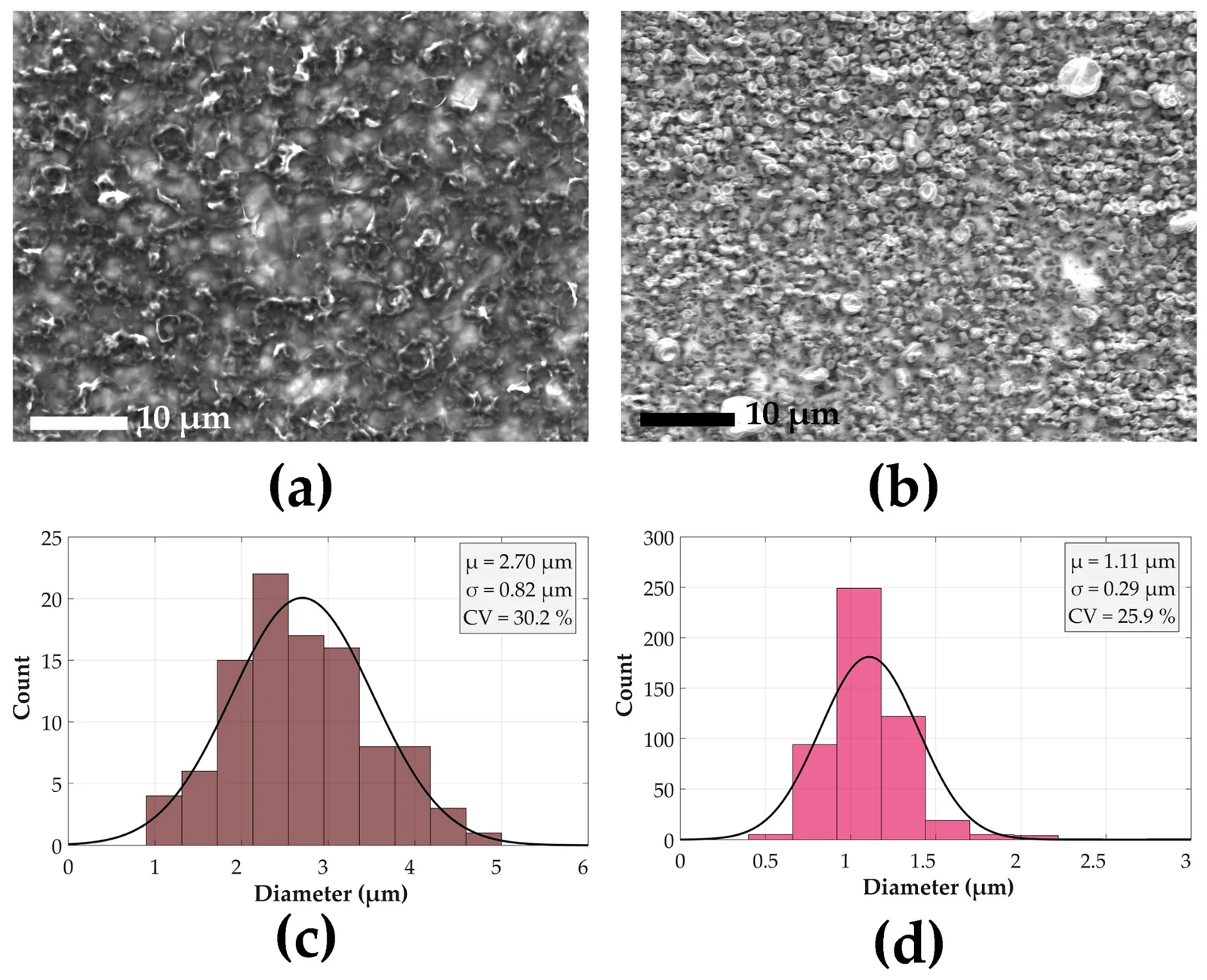
FE-SEM micrographs of ESMs deposited on one electrode of a 30 MHz QCM from (**a**) 0.3% *w*/*v* and (**b**) 5% *w*/*v* EC/EtOH solutions at 2000× magnification; corresponding particle-diameter distributions are shown in (**c**) and (**d**), respectively, with mean diameter μ, standard deviation σ, and coefficient of variation CV.

Morphology and average diameter of ESM sensing elements fabricated by electrospray are shown in Figure 6. The 0.3% *w*/*v* EC/EtOH solution formed spread, concave structures on the QCM electrode (Figure 6a) with an average size of 2.7 ± 0.8 μm and a coefficient of variation of 30% (Figure 6c). At 5% *w*/*v* EC/EtOH (Figure 6b), red blood cell-like concave structures were also observed, with an average size of 1.1 ± 0.3 μm and a CV of 26% (Figure 6d). Meanwhile, the red blood cell morphology can be attributed to forces in the electrospray process, as droplets split during their journey from the needle to the collector. At some point, droplets reach a critical radius where electrical stress exceeds capillary stress (Coulombic fission), leading to their internal collapse [30].

#### 4.1.2. Fourier Transform Infrared Spectroscopy Analysis

**Figure 7:**
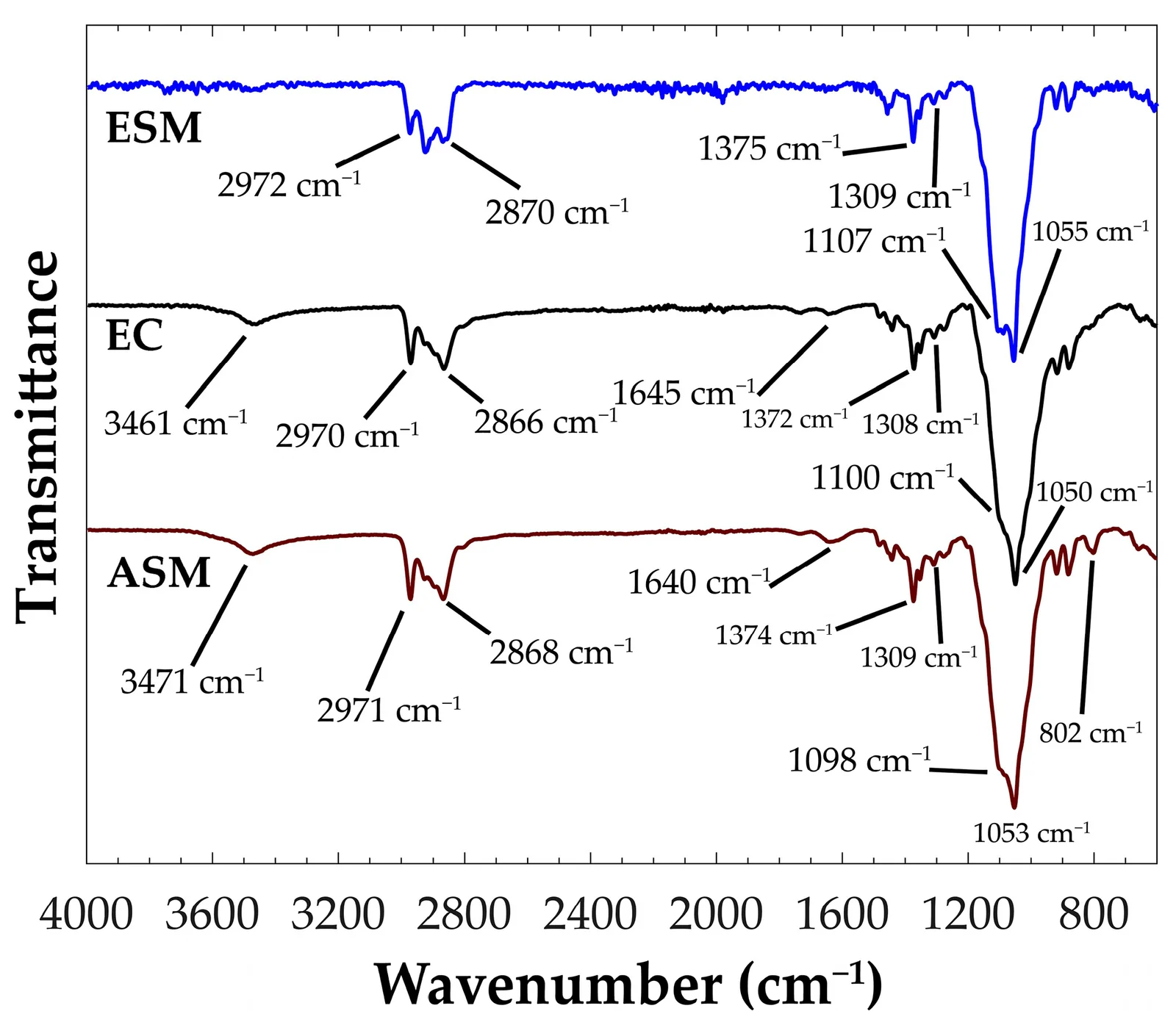
Normalized FTIR spectra of ethyl cellulose (EC), anti-solvent ethyl cellulose microparticles (ASMs), and ethyl cellulose electrospray microparticles (ESMs).

The transmittance FTIR spectra of the ethyl cellulose, the anti-solvent ethyl cellulose microparticles, and the electrospray microparticle sensing elements were normalized to avoid differences in sample thickness, morphology, and measurement conditions, shown in Figure 7, and the assigned bands are listed in Table 4. An O–H bending band is observed at 3471 and 3461 cm^−1^ for ASMs and EC, respectively, whereas this band is notably attenuated in the ESM sample. This attenuation suggests a reduced contribution of hydroxyl groups in the ESM sample, possibly associated with structural changes and water evaporation induced during the electrospraying fabrication process due to the applied high voltage. The reduction of O–H groups could help to have a higher affinity of ESMs toward hp relative to EtOH and EtOAc. The C–O bending bond appears at 1100, 1098, and 1107 cm^−1^ for EC, ASMs, and ESMs, respectively, getting progressively narrower in ASMs and ESMs compared with EC. The glycosidic C–O–C bond, which connects the pyranose rings in the polymer chain, appears at 1050 cm^−1^, 1053 cm^−1^, and 1055 cm^−1^ for EC, ASMs, and ESMs, respectively [63]. The bands at 1645 and 1640 cm^−1^ for EC and ASMs, respectively, were assigned to H–O–H bending of absorbed water [64]. In the ASM sample, the transmittance is higher, which is consistent with its hydrophilic nature. Finally, the vibrations of the pyranose ring appear at 1050–1055 cm^−1^ for all sensing elements, but were observed only at 802 cm^−1^ in the ASM sample, which could be linked to its specific morphology [65]. All these claims will be further addressed in more detail in Section 4.2.

### 4.2. QCM Sensor Steady-State Response and Sensitivity

**Figure 8:**
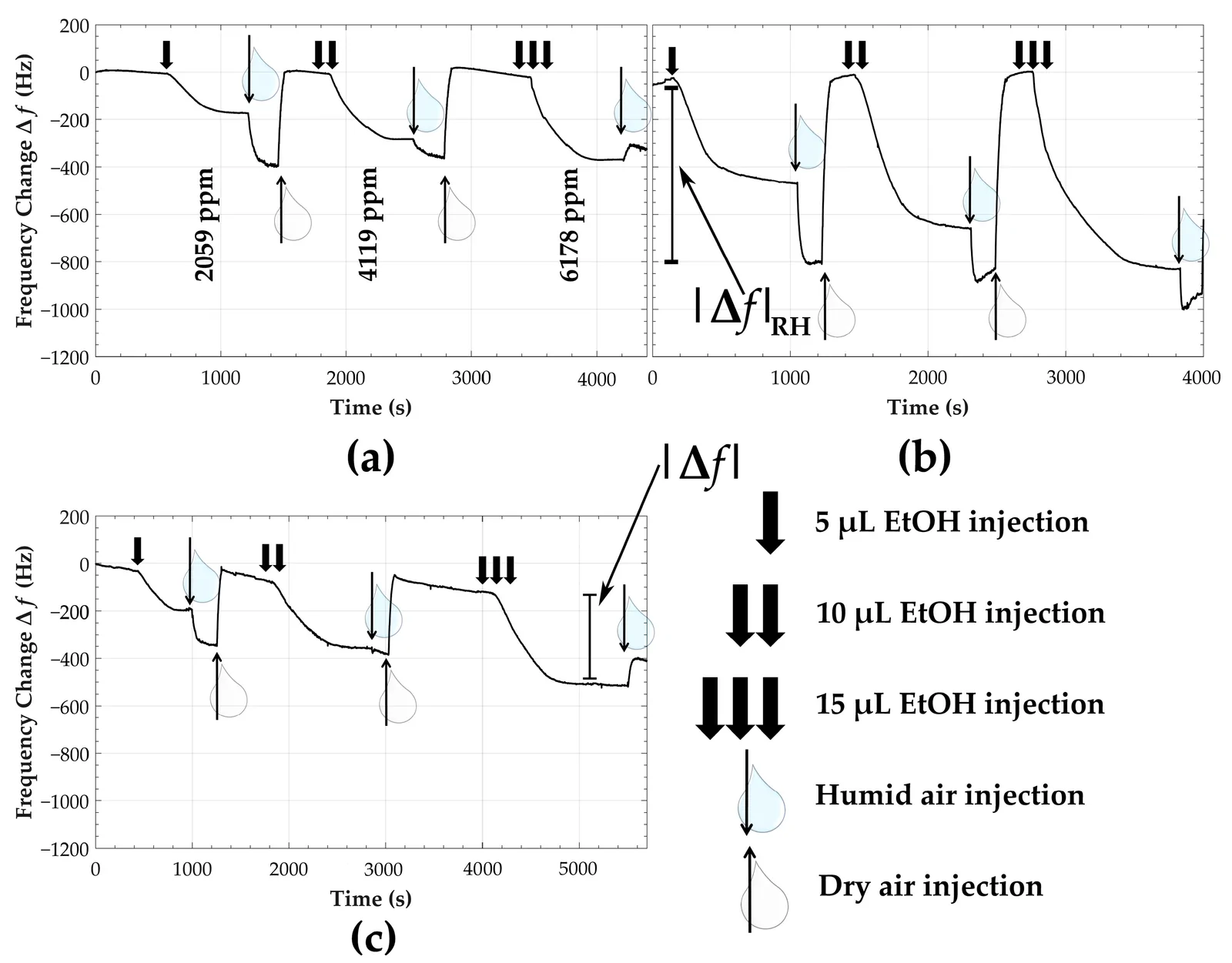
Oscillation curves of QCM sensors: (**a**) F214 (CF), (**b**) M250 (ASM), and (**c**) E223 (ESM) towards 2059, 4119, and 6178 ppm of EtOH, where |Δf| denotes the sensor’s response to EtOH.

Figure 8 shows the responses of sensors F214, M250, and E223 to EtOH at 2059, 4119, and 6178 ppm (5, 10, and 15 μL, respectively). The M250 sensor with self-assembled ASMs exhibited a larger |Δf| than F214 and E223, indicating a higher surface hydroxyl density and enhanced interaction with water. FTIR spectra suggest that ethyl-ether groups are encapsulated in ASMs, while the hydroxyl groups remain exposed. The increased |Δf| correlates with the larger surface area of micro/nanostructured particles. In contrast, F214 likely exposes both hydroxyl and ethoxy groups, whereas the E223 sensor response aligns with its FTIR.

**Figure 9:**
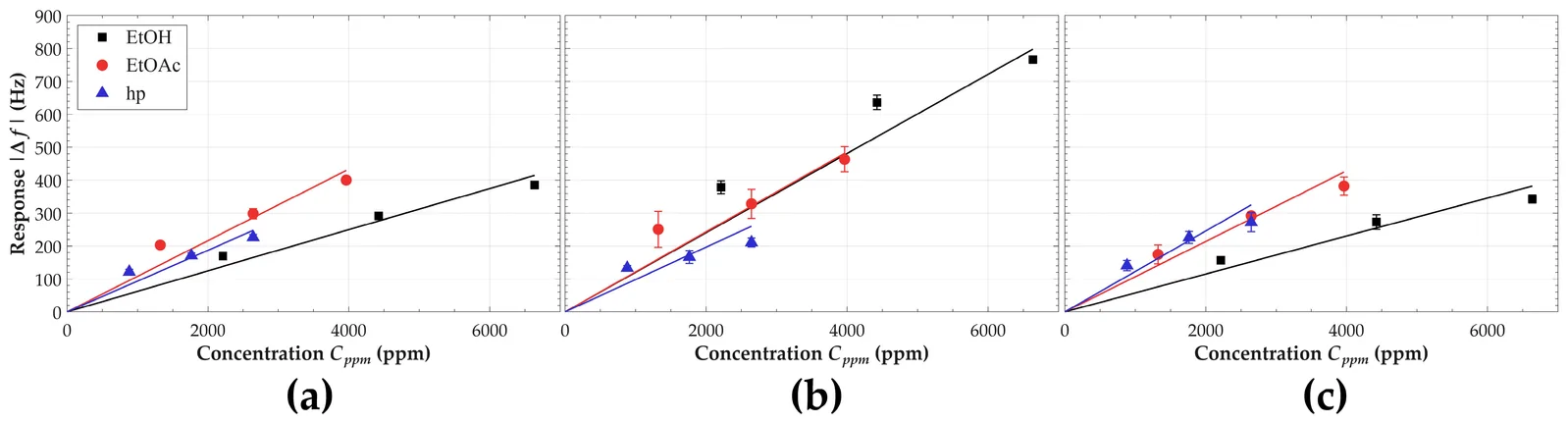
Average response of QCM sensors. (**a**) F214 (CF), (**b**) M250 (ASM), and (**c**) E223 (ESM) towards all three VOCs at different concentrations in ppm, as expressed in Table 3.

Figure 9 shows the average sensor response to VOC concentrations, with linear fits yielding $R^{2}$ > 0.96 (except for M78 for EtOAc), demonstrating data reliability. The slope, with units of Hz/ppm (Table 5, Table 6 and Table 7), indicates sensitivity. ASMs, with more exposed hydroxyl groups, responded more to polar VOCs like EtOH and EtOAc but less to non-polar VOCs (hp). The ESM sensor E223 showed higher sensitivity to hp, consistent with its surface rich in ethoxy groups that favor non-polar interactions, as supported by FTIR data showing no hydroxyl bands. CFs, as in sensor F214, exhibit intermediate behavior, indicating both hydroxyl and ethoxy groups.

**Figure 10:**
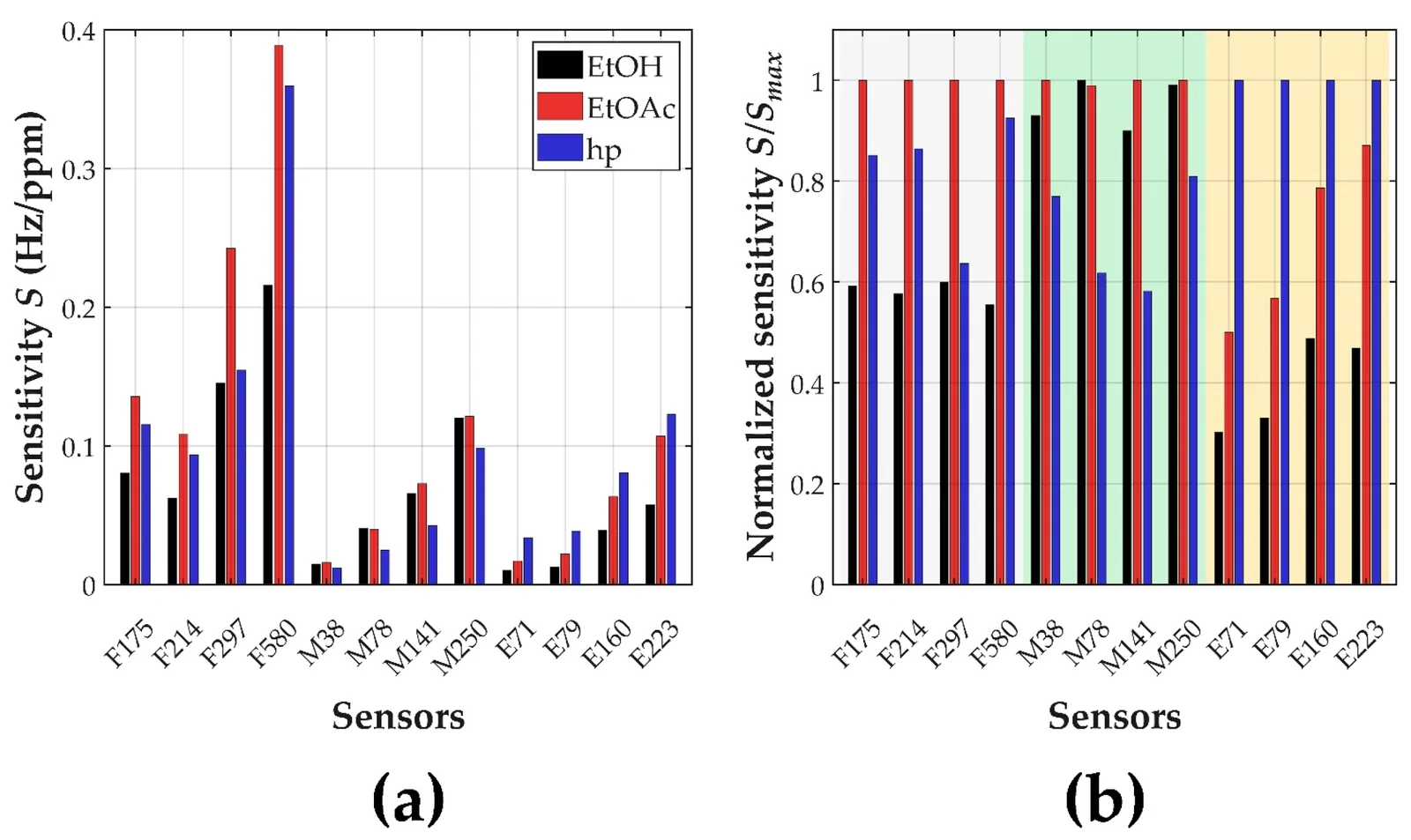
(**a**) Sensitivity S of the QCM sensors towards EtOH, EtOAc, and hp, being the slope of the linear fittings shown in Table 5, Table 6 and Table 7. (**b**) Sensitivity normalized to the maximum sensitivity of each sensor, *S*/*S*_max_.

Figure 10 shows the sensitivity S of QCM sensors to EtOH, EtOAc, and hp and the normalized sensitivity $S/S_{\max}$ to the maximum sensitivity of each sensor, allowing direct comparison of the relative sensitivity patterns, independent of the effective thickness of the QCM sensors. ASM sensors are more sensitive to EtOH and EtOAc, while the CF shows higher sensitivity to EtOAc, and the ESM displays higher sensitivity to hp, a nonpolar compound. Intermolecular forces between VOCs and the sensing element are crucial. ASMs expose more hydroxyl groups, enabling hydrogen bonding with EtOH and EtOAc, which disappears after purging the inside of the measurement chamber. EtOH interacts more due to its smaller size, but the carboxyl group of EtOAc, with a higher dipole moment and larger molar mass, causes a stronger interaction, slightly increasing sensitivity S to EtOAc. However, EtOH shows a similar sensitivity due to its size advantage. Meanwhile, nonpolar hp interacts weakly with ethoxy groups or polymer chains via London dispersion forces. Its frequency decrease mainly stems from its higher molar mass, the largest among the studied VOCs.

**Figure 11:**
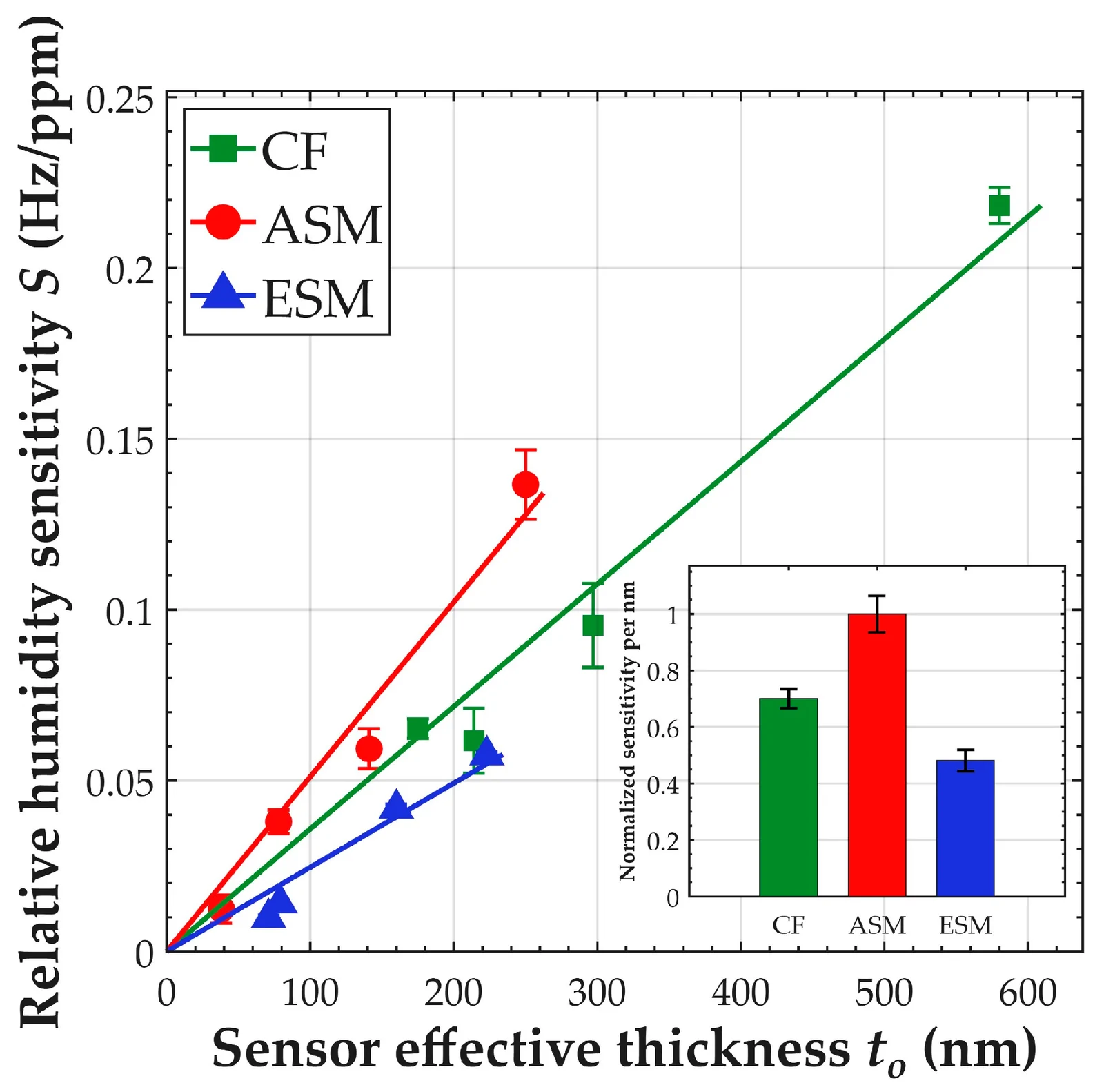
Sensitivity to relative humidity $S_{\mathrm{RH}}$ as a function of the sensor effective thickness. Inset: normalized sensitivity per nm per sensor type, which is the slope of the linear fittings (see Table 8).

The sensitivity of our sensors to relative humidity was estimated from their associated change in frequency denoted as $|\Delta f{|}_{\mathrm{RH}}$ in Figure 8, from the 20% to 45% increase in relative humidity during the purging process. From the Antoine equation and Antoine coefficients for water [51], we estimated the saturation vapor pressure of water to be 2344 Pa at 20 °C. Therefore, the partial vapor pressures of water at 20% and 45% relative humidity are 469 and 1055 Pa, respectively. Dividing by the total pressure of air, the standard atmospheric pressure of 101,325 Pa, and multiplying both values by $10^{6}$, we obtained that 20% relative humidity corresponds to 4627 ppm and 45% relative humidity to 10,410 ppm at 20 °C. Then, the average $|\Delta f{|}_{\mathrm{RH}}$ of each sensor was estimated from the response curves such as those shown in Figure 10. Assuming a linear relationship between $|\Delta f{|}_{\mathrm{RH}}$ in Hz and ppm concentration of water vapor $C_{\mathrm{ppm}}$, similar to the linear fittings of Table 5, Table 6 and Table 7, we obtained Equation (10),

(10) $$|\Delta f{|}_{\mathrm{RH}}=S_{\mathrm{RH}}C_{\mathrm{ppm}},$$

where $S_{\mathrm{RH}}$ represents the sensitivity to relative humidity, with Hz/ppm units. Thus, the average $|\Delta f{|}_{\mathrm{RH}}$ per sensor was divided by the increment in $C_{\mathrm{ppm}}$ from 20% to 45% relative humidity, 5783 ppm, producing the sensitivities $S_{\mathrm{RH}}$ shown in Figure 11 as a function of effective thickness in nm. Linear fittings per sensor type were performed, and the results are shown in Table 8. The inset in Figure 11 shows the normalized sensitivity per nm per sensor type, showing that CF and ESM sensors are 30% and 52% less sensitive to relative humidity than ASM sensors, respectively. This validates that ASM sensors are the most sensitive to relative humidity, while ESM sensors are the least sensitive.

The Hansen solubility parameters (HSP) in Table 1 describe VOC and ASM interactions, based on cohesive energy components: dispersion $\delta_{D}$, polar $\delta_{p}$, and hydrogen bonding $\delta_{H}$. Their squared sum gives the total solubility parameter $\delta_{t}$. While $\delta_{t}$ alone does not determine affinity, similarity across all three HSPs does. Among the VOCs studied, the HSPs of EtOAc are closest to EC, explaining its stronger affinity. The sensitivity of the ASM to EtOH nearly matches that of EtOAc due to EtOH’s larger surface area and smaller molecular size, enhancing interaction.

CFs, with 48% ethoxy content, combine features of the other sensing elements by exposing hydroxyl and ethoxy groups, enabling VOC interactions with both. Sensitivity variation among VOCs depends on molecular mass and intermolecular forces: EtOAc, with a carboxyl group, interacts more strongly with hydroxyl groups than EtOH and engages ethoxy groups due to lower polarity. The smaller size of the EtOH molecule increases interactions, but its higher polarity mainly limits binding to hydroxyl groups. Non-polar hp interacts only with ethoxy groups via London dispersion and Van der Waals forces; its higher molar mass produces a greater steady-state response, making the CF, on average, up to 42% more sensitive to hp than to EtOH.

In contrast, CFs show higher sensitivity than ASMs and ESMs at similar effective thicknesses $t_{o}$ due to more uniform electrode coverage. However, ASMs and ESMs leave partial coverage, with ASMs affected by the coffee-ring effect, in which particles gather at droplet edges [62]. Smaller ASMs increase surface area and VOC interaction, but sensitivity is limited by the non-uniform mass sensitivity of the QCM, which peaks at the electrode center and decays exponentially toward the edges [50], especially as smaller ASMs accumulate at the edges. Coating material deposited outside the electrode region does not contribute, as only the active area $A_{s}$ deforms at the fundamental frequency.

### 4.3. Principal Component Analysis, Mahalanobis Distance, and Support Vector Machine Algorithm for Classification

**Figure 12:**
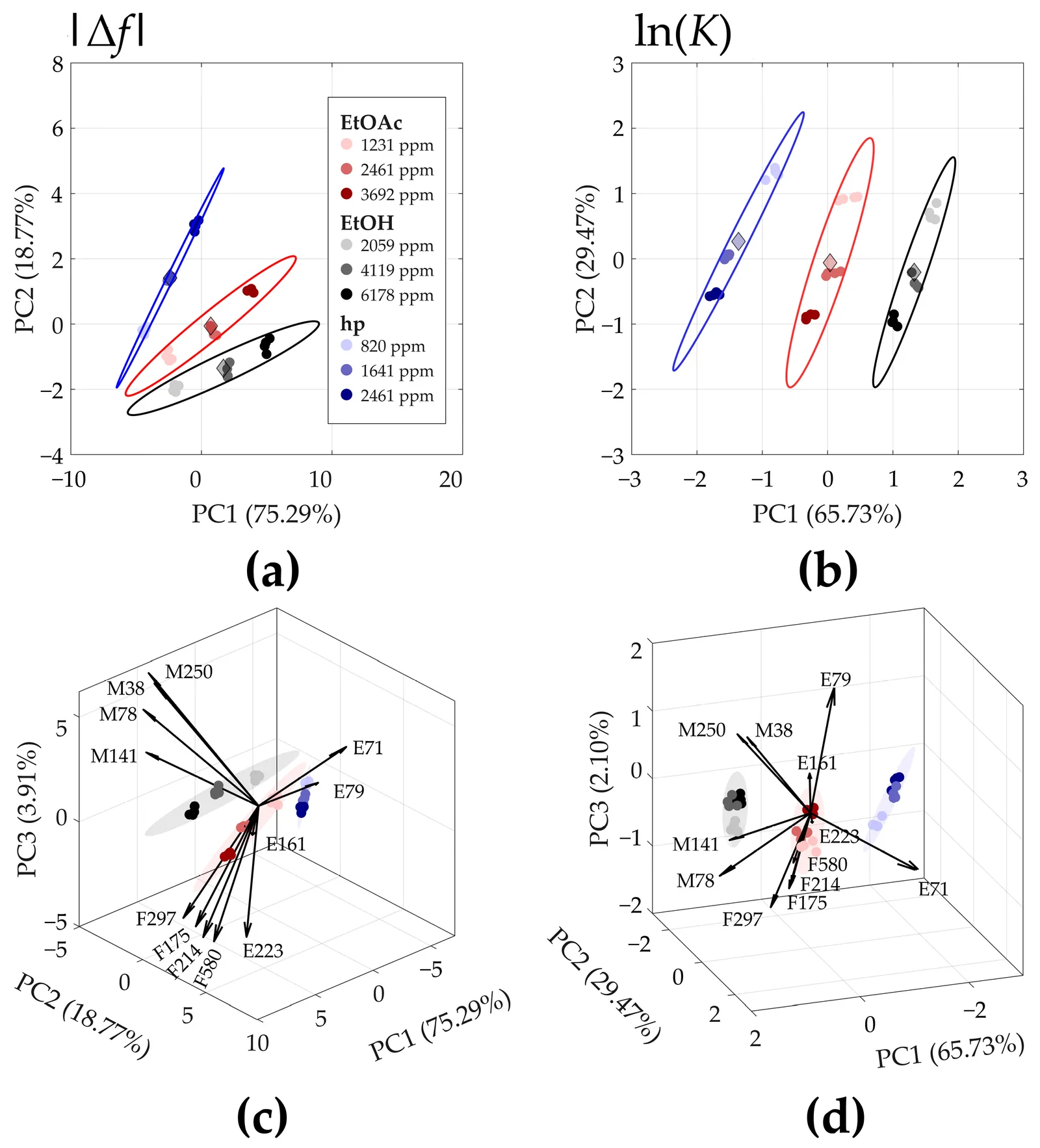
Principal component analysis of the QCM sensor response |Δf| (left) and ln(K) (right): (**a**,**b**) using 2 PCs; (**c**,**d**) are the corresponding biplots with 3 PCs.

Principal component analysis (PCA) was applied to the steady-state response |Δf| of the 12 sensors and to the partition coefficients ln(K) of the response, as per Equation (9), to reduce dimensionality. PCA identifies directions (PCs) that maximize variance, expressed as linear combinations of sensor responses, with eigenvalues corresponding to the explained variance. Figure 12 shows the |Δf| and ln(K) clusters by VOC and concentration using PC1, PC2, and PC3, with 98% and 97% cumulative explained variance, respectively. With only the first 2 PCs, the accumulated variance was 94% and 95%, respectively. Ellipses and ellipsoids at the 95% the confidence level (2σ), based on the Mahalanobis distance (MD), perfectly enclose all |Δf| and ln(K) values per VOC. Standardizing |Δf| improved separation, while ln(K) required no scaling and provided the clearest discrimination among compounds and concentrations. Figure 12c,d correspond to the PCA biplots, showing sensor contributions in the principal component space. ESM sensors shift right along hp |Δf| and ln(K), providing further evidence of their hydrophobic nature. CF sensors exhibit amphiphilic behavior (Figure 10). Finally, ASM sensors directly point to EtOH |Δf| and ln(K), suggesting that more hydroxyl groups are exposed on their surface.

In PCA space, the intra- and inter-MDs between groups (compounds) were computed, using the centroid of each group (the rhombuses in the PCA graphs in Figure 12a,b) as the reference. Focusing on the |Δf| graph (Figure 12a), all measurements for EtOAc and hp are at average distances of 7.76σ and 18.5σ from the EtOH centroid, respectively. For EtOAc, all measurements corresponding to EtOH and hp are at average distances of 7.48σ and 11.5σ from the EtOAc centroid, respectively. For hp, all measurements corresponding to EtOH and EtOAc are at average distances of 66.9σ and 44.1σ from the hp centroid, respectively. For the ln(K) (Figure 12b), all measurements corresponding to EtOAc and hp are at average distances of 16.8σ and 35.7σ from the EtOH centroid, respectively. As for the EtOAc, all measurements corresponding to EtOH and hp are at average distances of 12.1σ and 13.6σ from the EtOAc centroid, respectively. For hp, all measurements corresponding to EtOH and EtOAc are at average distances of 45.4σ and 24.3σ from the hp centroid, respectively. All distances exceed 2σ, indicating that the 95% confidence ellipses classify the three compounds correctly. Then, the MD is a suitable classifier for our data.

**Figure 13:**
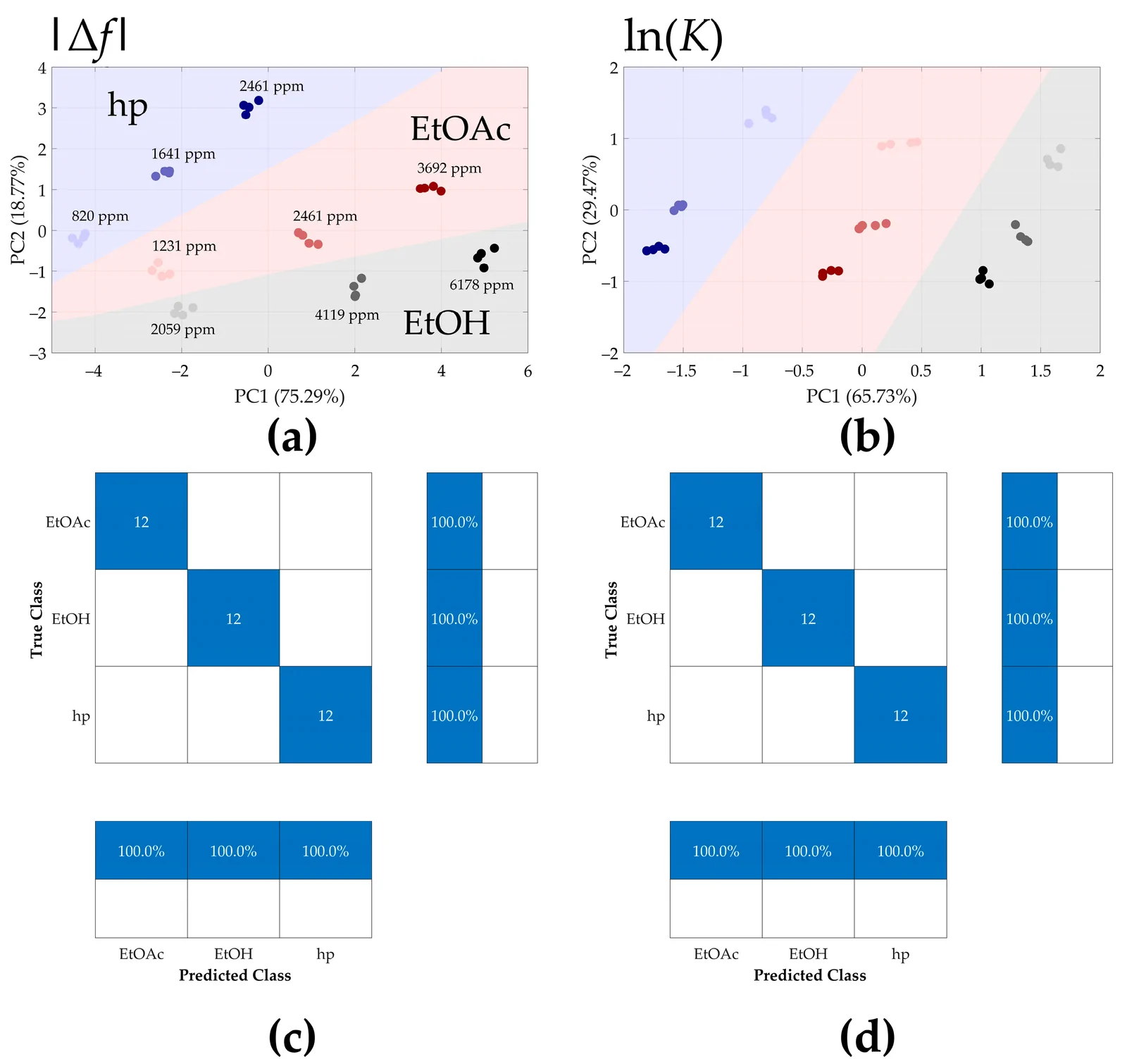
SVM decision boundaries in PCA space for the QCM sensor response: (**a**) |Δf| and (**b**) ln(K); (**c**,**d**) are the corresponding confusion matrices. Gray scale points correspond to EtOH measurements, red points are for EtOAc measurements, and blue points are for hp measurements. The different color shades correspond to the same concentrations shown in Figure 12a.

A multiclass SVM classifier with a linear kernel and internal standardization was trained with a 4-fold cross-validation method. This model classifies the three VOCs with high accuracy based on |Δf| and ln(K) data, both projected in the PCA space. The results are shown in Figure 13a and Figure 13b, respectively. Using ln(K) provides a simpler and more effective classification (Figure 13b). For |Δf|, at lower concentrations (Figure 13a), the classifier could be inaccurate regardless of data scaling, because the decision boundaries could converge. Although both |Δf| and ln(K) produce confusion matrices that show 100% accuracy and precision for both trained models, we can say that the classifier is better suited for ln(K), because the decision boundaries for every class are almost parallel to one another. In addition, ln(*K*), which is in itself a data scaling process of the |Δf|, provides clearer separation than the standardized |Δf|, also providing a broader separation between classes, as was quantified above by the Mahalanobis Distance.

### 4.4. Principal Component Regression and Partial Least Squares Regression for Concentration Prediction and Estimation of Limits of Detection

To build concentration-prediction models from the 12-sensor steady-state response data, we used principal component regression (PCR) and partial least squares regression (PLSR). PCR uses the principal components (PCs) from PCA to maximize variance, while PLSR, being supervised, derives latent variables (LVs) from sensor data that explain the greatest variance in the measured concentrations, reducing collinearity and improving model robustness. Because there are 12 measurements per VOC, four per concentration, PCR and PLSR models were evaluated with cross-validation as follows: for each VOC, one of the four measurements of each concentration was selected as validation measurements, while the remaining nine were used as training measurements for the PCR and PLSR models. In this 9/3 configuration (or 75% and 25%), a total of 64 different combinations were possible. This ensured that all three concentrations were present in both training and validation sets, for better accuracy. First, the PCR and PLSR models were trained using those 64 possible training sets, and the resulting 64 cross-validation root mean square errors (RMSEs), when comparing with the validation sets, were averaged, using one PC and LV, respectively. The same procedure was repeated using 2 PCs and LVs, until 8 PCs and LVs were employed.

**Figure 14:**
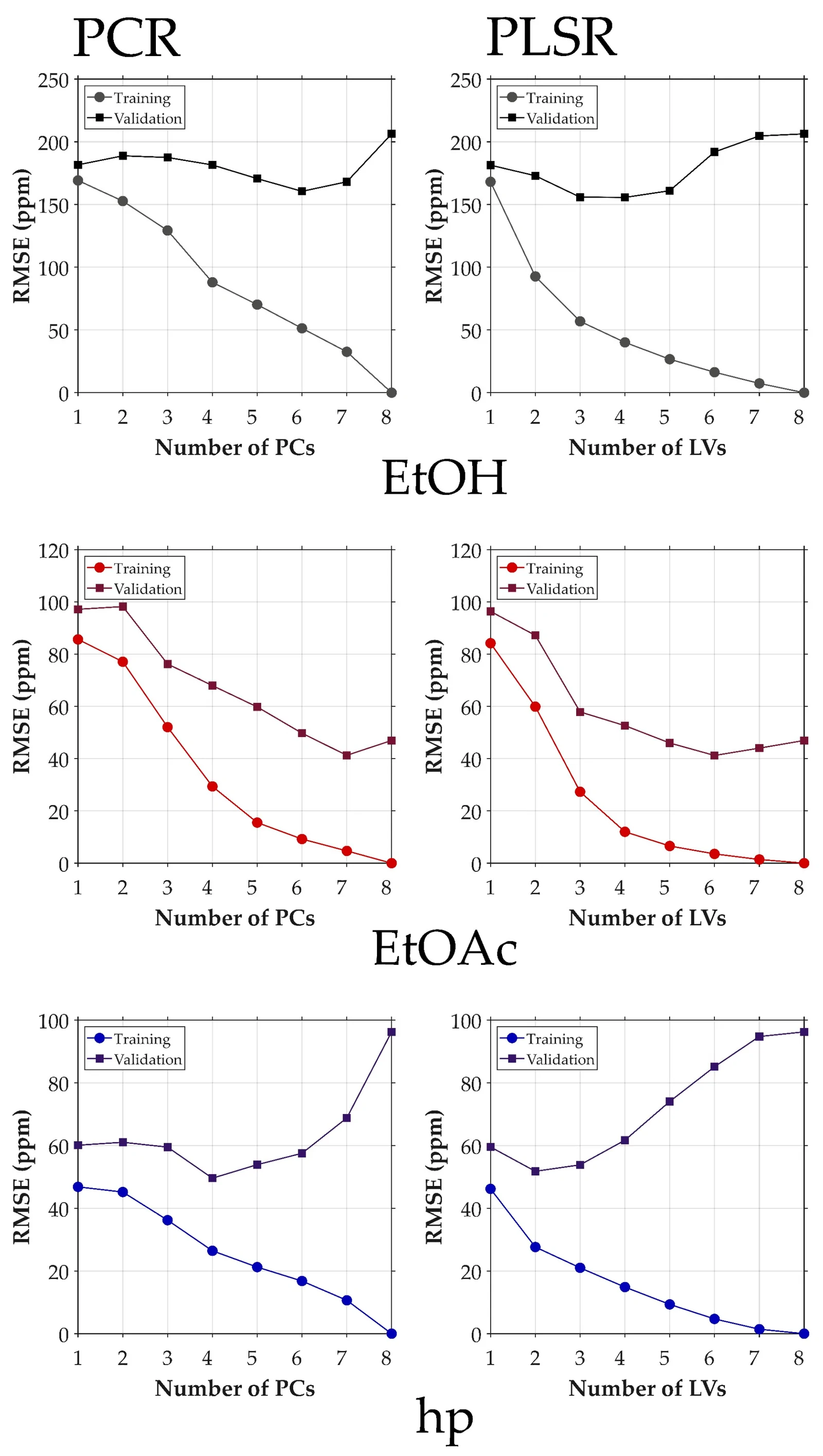
Cross-validation results: RMSE of the PCR (**left**) and PLSR (**right**) models with the training data set (circles) and the RMSE of the validation data set (squares) for EtOH (black), EtOAc (red), and hp (blue).

The average cross-validation RMSEs and overall model RMSEs were plotted against the number of PCs or LVs per compound to define the optimal number of these variables to avoid under- and overfitting in the PCR and PLSR models in Figure 14. The lowest cross-validation RMSE marks the optimal point before overfitting. Although the PCR model for EtOH indicates an optimal number of 6 PCs, the magnitude of the cross-validation RMSE does not change drastically from 1 to 7 PCs, so we can use a smaller number such as 3 PCs, while the PLSR model indicates that 3 LVs is the optimal number. For EtOAc, its PCR model states that 7 is the optimal number of PCs, while PLSR states that 6 LVs is the optimal number, but in both cases, the largest decrease occurs from the second to the third PC and LV; from there on the RMSE decreases in smaller steps; thus, 3 PCs and 3 LVs were considered a reasonable choice. For hp, the PCR and PLSR models state that 4 PCs and 2 LVs are the optimal numbers, respectively. However, in the PCR model the RMSE is stable in the first 3 PCs, and the RMSEs in the second and third LVs of the PLSR model do not change significantly. Thus, for the calibration curves, we fixed the number of PCs and LVs at 3 for all VOCs.

**Figure 15:**
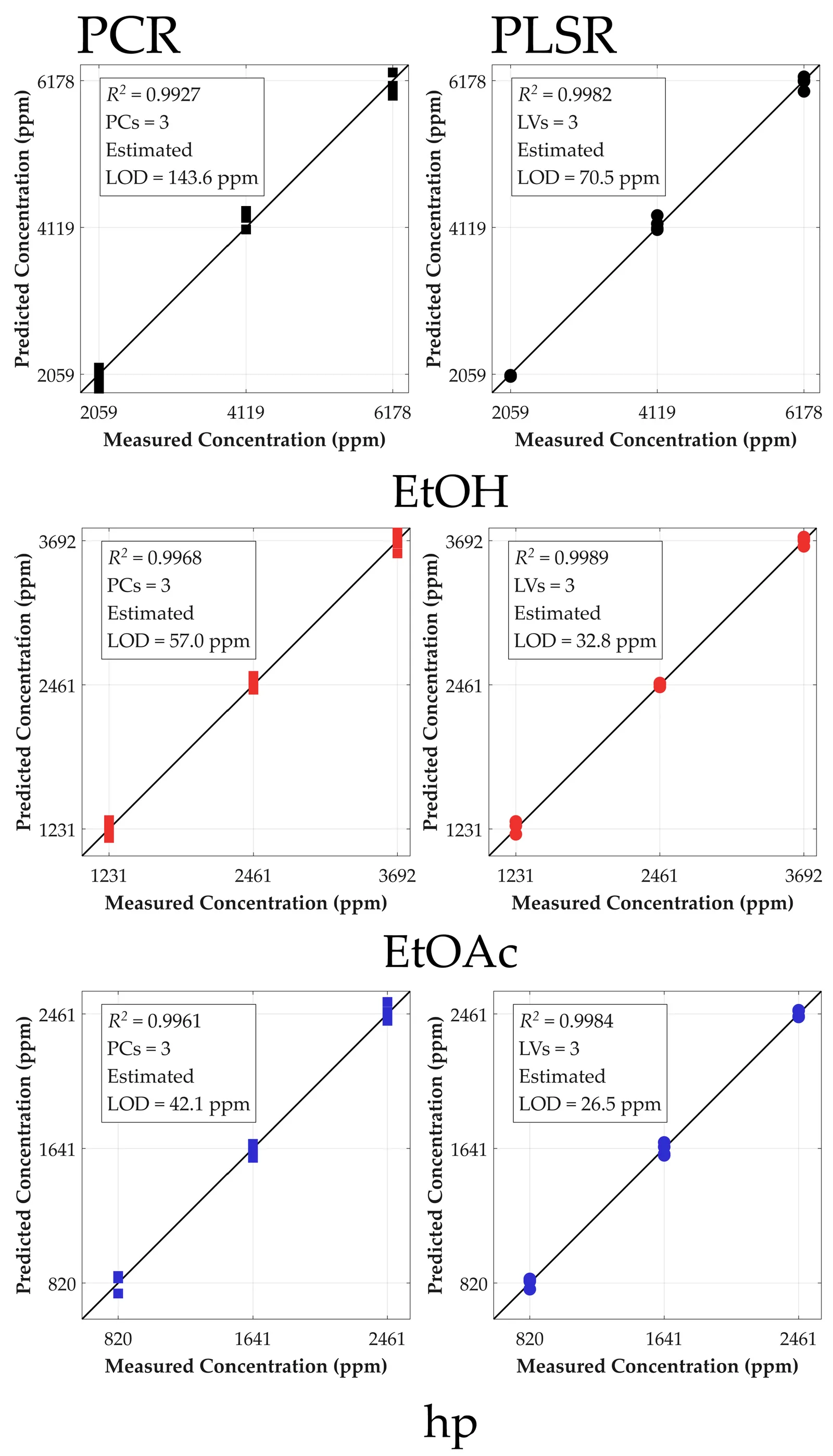
PCR (**left**) and PLSR (**right**) models using 3 PCs for PCR and 3 LVs for PLSR and the respective RMSE-estimated LOD values for EtOH (black), EtOAc (red), and hp (blue).

Figure 15 shows the calibration curves of the PCR and PLSR models for the three compounds using 3 PCs and 3 LVs, respectively, where the model-predicted concentrations are plotted against measured concentrations. The $R^{2}$ for each regression is shown and all values are above 0.99, indicating that the 12-sensor array can accurately predict the concentrations of the three VOCs. The limit of detection (LOD) is defined as the lowest concentration detectable with statistical confidence, related to the noise level in the data. Assuming SNR = 1, the estimated LODs per VOC of our sensor array were defined as the RMSEs of the predicted and measured concentrations of the calibration curves [42]. The RMSE-estimated LOD values are shown in Figure 15. The PCR and PLSR estimated LODs for EtOH are 143.6 ppm and 70.5 ppm; for EtOAc, these are 57.0 ppm and 32.8 ppm, and for hp, these are 42.1 ppm and 26.5 ppm, respectively. These results indicate that PLSR outperforms PCR by reducing the estimated LODs by up to 51%, using the same number of variables in each model. Also, these RMSE estimates correspond to 3.4%, 2.7%, and 3.2% of the lowest experimentally measured concentrations for EtOH, EtOAc, and hp, respectively, using PLSR models.

The estimated LOD values were substituted in the linear fitting equations of the sensor with the highest sensitivity towards all three VOCs, F580. The resulting estimated |Δf| values are shown in Table 9, along with the estimated instrumental LODs and corresponding frequency shifts |Δf|, calculated with consideration of the 1-Hz frequency-meter resolution. The estimated instrumental |Δf| values are approximately 10–31 times the resolution of the frequency-meter; thus, the estimated LODs can be measured with good precision with our single-polymer 30 MHz-QCM 12-sensor array.

## 5. Conclusions

A novel single-polymer 12-sensor array was developed based on 30 MHz QCMs coated with three ethyl cellulose microstructures: CF, ASM, and ESM, with four sensors per microstructure, for the detection of EtOH, EtOAc, and hp. Simple synthesis methods enabled the fabrication of CF, ASM, and ESM coatings, and SEM revealed distinct morphologies for each. CFs exhibited a continuous mesh of round holes with an average size of 1.13 ± 0.44 μm; ASMs, when deposited on the QCM electrodes, displayed average diameters of 60 ± 15 nm at the borders and 162 ± 52 nm in the center. ESMs had an average diameter of 1.1 ± 0.3 μm and exhibited collapsed-sphere or red-blood-cell-like morphologies, which may be associated with the Rayleigh dispersion phenomenon during electrospray. ASMs demonstrated sensitivity to polar compounds (EtOH, EtOAc), while ESMs were more sensitive to non-polar compounds (hp), and CF sensors exhibited a stronger response to all three compounds, particularly to EtOAc. These findings were supported by the steady-state response, the sensitivity patterns obtained from linear fittings of the response versus VOC concentration graphs, and FTIR analysis of functional groups. ASMs showed lower transmittance in the O–H and H–O–H bending bands, while ESMs exhibited higher transmittance in these bonds, consistent with their respective affinities for polar and nonpolar compounds. PCA of the response and ln(K) data successfully separated compounds and concentrations using only two principal components. The Mahalanobis distance and SVM classifier, applied to both response and ln(K) PCA graphs, distinguished the three VOCs, with ln(K) yielding clearer, more reliable decision boundaries and separation between all compounds. PCR and PLSR models predicted concentrations with $R^{2}$ > 0.99, where PLSR required fewer components, as determined by cross-validation. However, because cross-validation RMSEs did not vary substantially, we fixed the number of components at 3 for PCR and PLSR models for all compounds. The estimated LOD values with PCR were 143.6 ppm, 57 ppm, and 42.1 ppm for EtOH, EtOAc, and hp, respectively, while PLSR yielded estimated LODs of 70.5 ppm, 32.8 ppm, and 26.5 ppm, respectively. PLSR outperformed PCR by reducing estimated LODs to half of the original value. Tailoring ethyl cellulose microstructures by selecting solvents and deposition methods produced a versatile single-polymer 30 MHz QCM sensor array demonstrating VOC detection, classification, and concentration prediction under the tested conditions with estimated LODs in the tens-of-ppm range.

## Figures and Tables

**Table 1:** Physicochemical properties of ethyl cellulose and sensed VOCs [54,55,56,57,58,59,60,61].

Physicochemical Property	Ethanol	Ethyl Acetate	Heptane	Ethyl Cellulose *
Chemical formula	$C_{2}H_{6}O$	$C_{4}H_{8}O_{2}$	$C_{7}H_{16}$	$C_{20}H_{38}O_{11}$
Density (g/cm^3^)	0.789	0.902	0.684	1.140
Molar mass (g/mol)	46.07	88.11	100.21	454.51
Dipole moment (D)	1.69	1.81	0	—
*δ_D_* (MPa^1/2^)	15.8	15.8	15.3	17.9 *
*δ_H_* (MPa^1/2^)	19.4	7.2	0	3.9 *
*δ_P_* (MPa^1/2^)	8.8	5.3	0	4.3 *
*δ_t_* (MPa^1/2^)	26.5	18.2	15.3	18.8 *

* The EC solubility parameters are for the commercial grade ETHOCEL HE10, in which ‘10’ denotes the viscosity in cP [61].

**Table 2:** Sensor label, effective thickness $t_{o}$, sensor type, and needle-to-collector distance $d_{\mathrm{nc}}$.

Sensor Label	$t_{o}$ (nm)	Sensor Type	$d_{\mathrm{nc}}$ (cm)
F175	175	CF (F)	—
F214	214		
F297	297		
F580	580		
M38	38	ASM (M)	—
M78	78		
M141	141		
M250	250		
E71	71	ESM (E)	10
E79	79		
E160	160		5
E223	223		

**Table 3:** VOCs sensed by the 12 sensors and their concentrations in ppm inside the sensing chamber at T = 20 °C and RH = 20% determined by the injected volume $V_{v}$ and Equation (4).

$V_{v}$ (μL)	VOC Concentration (ppm)		
	EtOH	EtOAc	hp
5	2059	1231	820
10	4119	2461	1641
15	6178	3692	2461

**Table 4:** Infrared analysis of EC, ASMs, and ESMs based on [65].

Bond	Wavenumber (cm^−1^)		
	EC	ASM	ESM
O–H bending	3461	3471	—
C–H stretching	2970	2971	2972
C–H group	2866	2868	2870
H–O–H bending	1645	1640	—
H–C–H bending	1372	1374	1375
O–H–C bending	1308	1309	1309
C–O bending	1100	1098	1107
C–O–C bond (between rings)	1050	1053	1055
C–O–C bond (pyranose ring)	—	802	—

**Table 5:** Fitting equations of the response of the CF QCM sensors to EtOH, EtOAc, and hp.

Sensor	Equation for EtOH	$R^{2}$	Equation for EtOAc	$R^{2}$	Equation for hp	$R^{2}$
F175	$|\Delta f|=(0.0804\;\pm \;0.0088)C_{\mathrm{ppm}}$	0.9991	$|\Delta f|=(0.1358\;\pm \;0.0119)C_{\mathrm{ppm}}$	0.9995	$|\Delta f|=(0.1156\;\pm \;0.0184)C_{\mathrm{ppm}}$	0.9999
F214	$|\Delta f|=(0.0624\;\pm \;0.0037)C_{\mathrm{ppm}}$	0.9972	$|\Delta f|=(0.1083\;\pm \;0.0113)C_{\mathrm{ppm}}$	0.9998	$|\Delta f|=(0.0936\;\pm \;0.0099)C_{\mathrm{ppm}}$	0.9994
F297	$|\Delta f|=(0.1455\;\pm \;0.0100)C_{\mathrm{ppm}}$	0.9999	$|\Delta f|=(0.2425\;\pm \;0.0198)C_{\mathrm{ppm}}$	0.9993	$|\Delta f|=(0.1445\;\pm \;0.0106)C_{\mathrm{ppm}}$	0.9968
F580	$|\Delta f|=(0.2158\;\pm \;0.0145)C_{\mathrm{ppm}}$	0.9995	$|\Delta f|=(0.3886\;\pm \;0.0198)C_{\mathrm{ppm}}$	0.9983	$|\Delta f|=(0.3595\;\pm \;0.0359)C_{\mathrm{ppm}}$	0.9999

**Table 6:** Fitting equations of the response of the ASM QCM sensors to EtOH, EtOAc, and hp.

Sensor	Equation for EtOH	$R^{2}$	Equation for EtOAc	$R^{2}$	Equation for hp	$R^{2}$
M38	$|\Delta f|=(0.0147\;\pm \;0.0015)C_{\mathrm{ppm}}$	0.9681	$|\Delta f|=(0.0158\;\pm \;0.0016)C_{\mathrm{ppm}}$	0.9946	$|\Delta f|=(0.0121\;\pm \;0.0018)C_{\mathrm{ppm}}$	0.9993
M78	$|\Delta f|=(0.0403\;\pm \;0.0048)C_{\mathrm{ppm}}$	0.9935	$|\Delta f|=(0.0399\;\pm \;0.0079)C_{\mathrm{ppm}}$	0.6833	$|\Delta f|=(0.0249\;\pm \;0.0056)C_{\mathrm{ppm}}$	0.9049
M141	$|\Delta f|=(0.0656\;\pm \;0.0102)C_{\mathrm{ppm}}$	0.9917	$|\Delta f|=(0.0729\;\pm \;0.0043)C_{\mathrm{ppm}}$	0.9999	$|\Delta f|=(0.0424\;\pm \;0.0053)C_{\mathrm{ppm}}$	0.9724
M250	$|\Delta f|=(0.1202\;\pm \;0.0091)C_{\mathrm{ppm}}$	0.9824	$|\Delta f|=(0.1214\;\pm \;0.0100)C_{\mathrm{ppm}}$	0.9879	$|\Delta f|=(0.0983\;\pm \;0.0209)C_{\mathrm{ppm}}$	0.9968

**Table 7:** Fitting equations of the response of the ESM QCM sensors to EtOH, EtOAc, and hp.

Sensor	Equation for EtOH	$R^{2}$	Equation for EtOAc	$R^{2}$	Equation for hp	$R^{2}$
E71	$|\Delta f|=(0.0103\;\pm \;0.0005)C_{\mathrm{ppm}}$	0.9978	$|\Delta f|=(0.0169\;\pm \;0.0010)C_{\mathrm{ppm}}$	0.9993	$|\Delta f|=(0.0339\;\pm \;0.0012)C_{\mathrm{ppm}}$	0.9994
E79	$|\Delta f|=(0.0128\;\pm \;0.0010)C_{\mathrm{ppm}}$	0.9888	$|\Delta f|=(0.0220\;\pm \;0.0007)C_{\mathrm{ppm}}$	0.9977	$|\Delta f|=(0.0387\;\pm \;0.0010)C_{\mathrm{ppm}}$	0.9999
E160	$|\Delta f|=(0.0393\;\pm \;0.0014)C_{\mathrm{ppm}}$	0.9977	$|\Delta f|=(0.0633\;\pm \;0.0026)C_{\mathrm{ppm}}$	0.9996	$|\Delta f|=(0.0805\;\pm \;0.0026)C_{\mathrm{ppm}}$	0.9950
E223	$|\Delta f|=(0.0576\;\pm \;0.0059)C_{\mathrm{ppm}}$	0.9899	$|\Delta f|=(0.1070\;\pm \;0.0054)C_{\mathrm{ppm}}$	0.9899	$|\Delta f|=(0.1229\;\pm \;0.0137)C_{\mathrm{ppm}}$	0.9857

**Table 8:** Linear fitting equations of the sensitivity towards relative humidity of the sensors as a function of effective thickness from Figure 11.

Sensor Type	Linear Fitting Equation	$R^{2}$
CF	$|\Delta f|=0.00036\;\pm \;0.00002C_{\mathrm{ppm}}$	0.9716
ASM	$|\Delta f|=0.00051\;\pm \;0.00003C_{\mathrm{ppm}}$	0.9660
ESM	$|\Delta f|=0.00025\;\pm \;0.00002C_{\mathrm{ppm}}$	0.9365

**Table 9:** RMSE-estimated LODs in ppm of EtOH, EtOAc, and hp using PCR and PLSR.

VOC	Number of Components		Estimated LOD (ppm)		Estimated |Δf| (Hz)		Estimated Instrumental LOD (ppm)		Estimated Instrumental |Δf| (Hz)	
	PCR	PLSR	PCR	PLSR	PCR	PLSR	PCR	PLSR	PCR	PLSR
EtOH	3	3	143.6	70.5	30.9	15.2	144.1	69.6	31.0	15.0
EtOAc	3	3	57.0	32.8	22.2	12.7	56.5	33.6	22.0	13.0
hp	3	3	42.1	26.5	15.4	9.53	41.0	27.8	15.0	10.0

## Data Availability

The raw data supporting the conclusions of this article will be made available by the authors on request.
